# Little ecological divergence associated with speciation in two African rain forest tree genera

**DOI:** 10.1186/1471-2148-11-296

**Published:** 2011-10-11

**Authors:** Thomas LP Couvreur, Holly Porter-Morgan, Jan J Wieringa, Lars W Chatrou

**Affiliations:** 1 Institut de Recherche pour le Développement (IRD), UMR DIA-DE, DYNADIV researche group, 911, avenue Agropolis, BP 64501, F-34394 Montpellier cedex 5, France; 2 The New York Botanical Garden, 200th St. and Kazimiroff Blvd, Bronx, NY 10458-5126, USA; 3 LaGuardia Community College, City University of New York, 3110 Thomson Avenue, Long Island City, NY, USA; 4 Netherlands Centre for Biodiversity Naturalis (section NHN), Wageningen branch, and Wageningen University, Biosystematics group, Generaal Foulkesweg 37, 6703 BL Wageningen, The Netherlands; 5 Wageningen University, Biosystematics group, Droevendaalsesteeg 1, 6708 PB Wageningen, The Netherlands

## Abstract

**Background:**

The tropical rain forests (TRF) of Africa are the second largest block of this biome after the Amazon and exhibit high levels of plant endemism and diversity. Two main hypotheses have been advanced to explain speciation processes that have led to this high level of biodiversity: allopatric speciation linked to geographic isolation and ecological speciation linked to ecological gradients. Both these hypotheses rely on ecology: in the former conservation of ecological niches through time is implied, while in the latter adaptation via selection to alternative ecological niches would be a prerequisite. Here, we investigate the role of ecology in explaining present day species diversity in African TRF using a species level phylogeny and ecological niche modeling of two predominantly restricted TRF tree genera, *Isolona *and *Monodora *(Annonaceae). Both these genera, with 20 and 14 species, respectively, are widely distributed in African TRFs, with a few species occurring in slightly less humid regions such as in East Africa.

**Results:**

A total of 11 sister species pairs were identified most of them occurring in allopatry or with little geographical overlap. Our results provide a mixed answer on the role of ecology in speciation. Although no sister species have identical niches, just under half of the tests suggest that sister species do have more similar niches than expected by chance. PCA analyses also support little ecological differences between sister species. Most speciation events within both genera predate the Pleistocene, occurring during the Late Miocene and Pliocene periods.

**Conclusions:**

Ecology is almost always involved in speciation, however, it would seem to have had a little role in species generation within *Isolona *and *Monodora *at the scale analyzed here. This is consistent with the geographical speciation model for TRF diversification. These results contrast to other studies for non-TRF plant species where ecological speciation was found to be an important factor of diversification. The Pliocene period appears to be a vital time in the generation of African TRF diversity, whereas Pleistocene climatic fluctuations have had a smaller role on speciation than previously thought.

Ecological niche modeling, species level phylogeny, ecological speciation, African tropics, *Isolona*, *Monodora*, Annonaceae

## Background

The tropical rain forest (TRF) biome covers just ~7% of land but harbors over half of the planet's terrestrial biodiversity. The TRF of Africa represents the second largest extent of this biome after the Amazon basin, and contains high levels of species diversity and especially endemicity [1]. Two main African rain forest blocks exist. The most widespread one corresponds to the Guineo-Congolian floristic region [2], which extends almost continuously (expect for a break at the Dahomey gap in Togo and Benin) from West Africa into the Congo basin and east of the Democratic Republic of Congo and Uganda. The East African rain forests are concentrated on a smaller and patchier surface extending from Kenya to southern Mozambique along the coast and the Eastern Arcs [3,4]. This latter block contains one of the highest concentrations of endemic species on Earth [5].

Understanding the evolutionary processes responsible for high species richness of TRF has been a major focus of evolutionary biology [6], although most studies have focused on the Amazon and Australian rain forests. Two main hypotheses have been advanced to explain speciation processes that have led to high levels of biodiversity in African TRF [7,8]: 1) geographic isolation between populations restricted to rain forest patches resulting in allopatric speciation (e.g. the refuge model, mountain speciation model, riverine barrier model [9-13]); 2) divergent selection related to ecological gradients where genetic and/or geographic isolation is minor [7,14]. Ecology is central to the processes of divergence and speciation [15,16] but in the two speciation models above (geographic isolation vs. divergent selection) ecology plays contrasting roles [17]. In the former, it would be expected that the variables that make up a niche have low rates of change over a phylogeny (niche conservatism [18,19] or niche stasis [20]). This pattern would be predicted when taxa that are restricted to rain forest patches have been tracking the geographical distribution of this biome, being unable to adapt to changing conditions. In contrast, in the case of divergent selection, ecological divergence and adaptation to new niches would be a fundamental prerequisite [7], and the variables comprising species' niches would be expected to deviate less from a random distribution over a phylogeny than in the case of geographic isolation. For example, adaptation to more arid environments since the Miocene has been suggested to be a driver of diversification in some African plant genera [21]. Thus, a possible first step in unraveling TRF diversification in Africa is to understand the role of ecology in explaining present day diversity.

New developments in ecological niche modeling (ENM) or species distribution modeling have provided important advances in the understanding of species distribution [22], as well as in the study of ecological speciation [e.g. for plants [23], [24], [25]]. These methods combine data about the known distribution of a species with climatic and other relevant variables to identify those environmental conditions that are suitable for that species and predict its potential distribution. Such methods allow for a precise quantification of ecological parameters specific to each species, which in turn enables the use of statistical tests to answer questions about niche differentiation or similarity [26,27].

Here, we used an integrative approach including a dated molecular phylogeny [28,29] and ENM to investigate the evolutionary ecology of two African TRF genera *Isolona *and *Monodora *(Annonaceae). Both genera contain small to large trees largely distributed across the African rain forests from West/Central Africa to East Africa. A recent monograph of both genera provides information about species delimitation as well as their distribution [28]. *Isolona *contains 20 species, 5 of them endemic to Madagascar, while *Monodora *contains 14 species and is absent from Madagascar [28]. A large number of species grow in lowland rain forests, but a few occur in montane areas (above 1200 m), as well as in less humid regions growing in thicket or woodland such as in Malawi and northern South Africa. Both genera have been recovered as sister with maximum support nested within a large clade of African genera [30]. Moreover, the presence of phylogenetic signal of certain climatic variables identified within *Monodora *has been previously suggested but not explicitly tested [31]. Finally, Hutchinson underlined [32] that the ecological niche of species is multi dimensional. For this study we shall use climatic data for Africa as this is available for all species used in this study, in contrast to other ecological data for which we have very spars records (pollination biology, biological interactions, etc.). Moreover, bioclimatic models appear suitable to generate predictions of species ecological requirements at the macro-scale studied here (continental distribution of species) [33].

The goal of this study was to understand the role of ecology in the speciation and distribution of both genera. We identified sister species and tested for niche similarity and for phylogenetic signal of different climatic variables within the clade. Specifically we addressed the following questions: Do sister species have similar ecological niches? Can we detect a phylogenetic signal of environmental variables?

## Results

### Phylogeny

**Figure 1:**
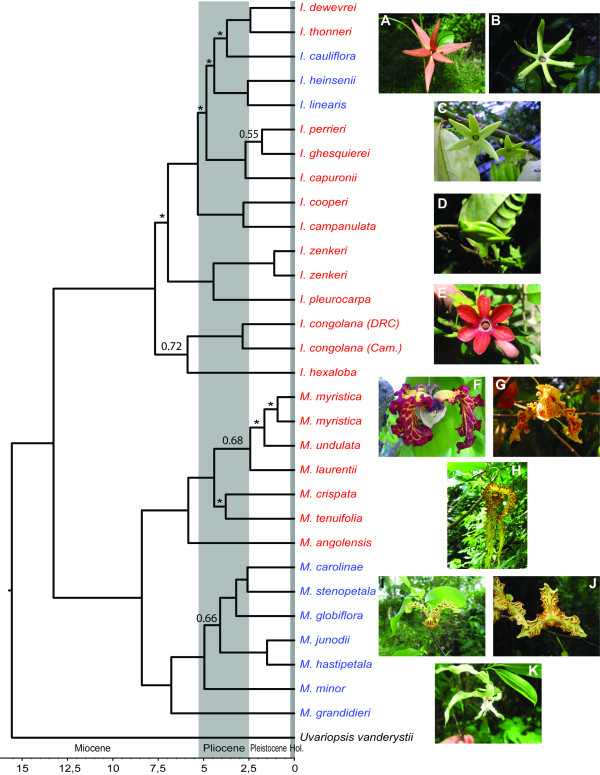
**Tempo of speciation in *Isolona *and *Monodora***. Maximum clade credibility chronogram, with nodes represented by their mean ages estimated from 9000 posterior trees and under a relaxed lognormal uncorrelated molecular clock assumption. Values at nodes represent posterior probabilities (PP) of the BEAST analysis. Nodes without values have PP of 0.8-1. Asterisks indicate nodes with less than 0.5 PP. Taxa in blue: West/Central Africa; Taxa in red: east Africa. A: *Isolona cauliflora*; B: *I. heinsenii*; C: *I. cooperi*; D: *I. zenkeri*; E: *I. hexaloba*; F: *Monodora myristica*; G: *M. undulata*; H: *M. crispata*; I: *M. carolinae*; J: *M. globiflora*; K: *M. hasipetala*. (Photos: TLP Couvreur, except G, C. Jongkind). Hol.: Holocene. DRC: Democratic Republic of Congo. Cam: Cameroon.

Using the molecular phylogenies published by [28,29] we identified a total of 11 species pairs (Figure 1) generally based on strong support values. No comparisons were undertaken within the monophyletic Malagasy clade because the focus of this study was directed towards mainland Africa. In the cases where support values were low comparisons were undertaken on species that were grouped together in the different prior phylogenetic analyses (e.g. BEAST, MrBayes). Morphology and palynology provide little information on species relationships when compared to molecular phylogenies, especially in *Monodora *[28,34]. *Monodora crispata *and *M. tenuifolia *were considered as sister albeit with low support because these two species were also recovered as sister (with low support) in other analyses [see [28]]. In one case, relationships between three species (*M. myristica*, *M. undulata *and *M. laurentii*) were unresolved and thus comparisons were undertaken between all possible pairs (four pairwise comparisons). Within *Isolona*, four species of mainland Africa were not sampled and their exact placement remains unknown. Even though strong support for some sister species relationships are lacking, the phylogeny of Couvreur et al. [28,29] provides the best hypothesis of species relationship to date.

Mean divergence ages (Table 1) for all sister species ranged from on average 1.6 to 6 million years ago (Ma). In some cases, estimation of the 95% highest posterior density confidence intervals was not possible given the low node support. Finally, most sister species pairs occurred in allopatry or had little geographic range overlap (Table 1). Calculations indicated that species pairs varied from no overlap (0% in two cases) to 89.3% overlap (one case, see discussion).

**Table 1:** Sister species characteristics in *Isolona *and *Monodora *as well as results from the PCA and, the similarity and background tests.

				Mann-Whitney U					Identity test		Background test (1)	
**Species pair**	**% geographical overlap**	**Smaller Range species**	**MRCA age (95% confidence interval)**	**PC1**	**PC2**	**PC3**	**D**	**I**	**D**	**I**	**D**	**I**

***Isolona***												
*pleurocarpa-zenkeri*	19.6	*pleurocarpa*	4.5 (1.4-7.9)	*	NS	***	0,25	0,50	***	***	more */more *	more */more **
*dewevrei-thonneri*	8.9	*dewevrei*	2.5 (0.4-4.8)	***	NS	***	0,41	0,63	***	***	NS/NS	NS/NS
*heinsenii-linearis*	32	*linearis*	2.6 (0.7-4.8)	*	NS	NS	0,40	0,62	***	***	less ***/NS	less ***/more *
*campanulata-cooperi*	16	*cooperi*	2.8 (0.9-5.2)	NS	*	NS	0,53	0,69	***	***	NS/more *	NS/NS
*congolana-hexaloba*	12.6	*congolana*	6 (2.7-9.8)	***	**	**	0,44	0,60	***	***	NS/NS	NS/NS
***Monodora***												
*carolinae-stenopetala*	0	no overlap	2.6 (0.9-4.4)	NS	**	**	0,21	0,49	***	***	*** more/NS	*** more/NS
*hastipetala-junodii*	89.3	*hastipetala*	1.5 (0.2-3.2)	NS	*	NS	0,05	0,35	***	***	less */less ***	less */less ***
*laurentii-myristica*	14.3	*laurentii*	2.4 (0.6-4.8)	*	NS	NS	0,55	0,70	***	***	more ***/more ***	more ***/more ***
*myristica-undulata*	35.7	*undulata*	1.6 (NE)	**	***	***	0,43	0,64	***	***	NS/NS	more */NS
*crispata-tenuifolia*	43	*crispata*	3.8 (NE)	NS	***	NS	0,46	0,66	***	***	more ***/more *	more ***/more *
*laurentii-undulata*	0	no overlap	2.4 (0.6-4.8)	NS	**	**	0,23	0,50	***	***	more ***/more ***	more ***/more *

NE = not estimated

*** P < 0,001

** P < 0,01

* P < 0,05

NS not significant

1) the first value corresponds to the test of the first species vs the background of second, and then vice versa

### Bioclim variables

**Figure 2:**
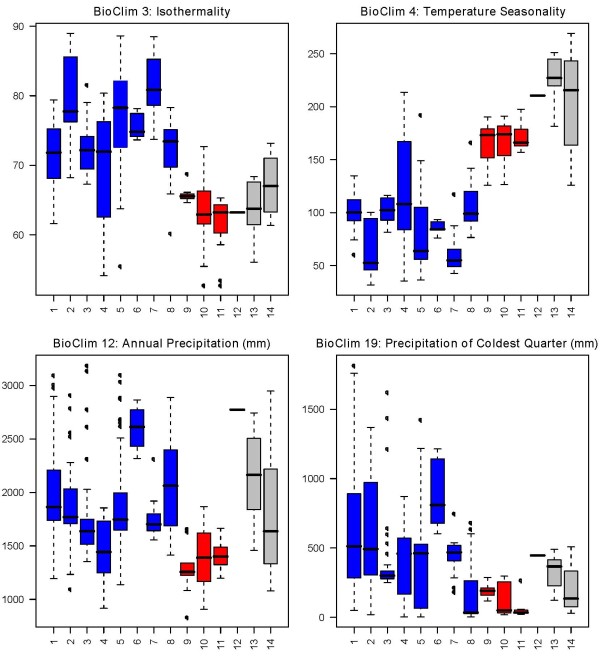
**Climatic variation in species of *Isolona***. Only species sampled in the molecular phylogeny are represented. Blue: West/Central African species; Red: East African species; grey: Malgasy species. West/Central African species: 1: *Isolona congolana; *2: *I. hexaloba; *3: *I. pleurocarpa; *4: *I. zenkeri; *5: *I. campanulata; *6: *I. cooperi; *7: *I. dewevrei; *8: *I. thonneri; *9: *I. cauliflora*. East African species: 10: *I. heinsenii; *11: *I. linearis*. Malagasy species: 12: *I. capuroni; *13: *I. ghesquierei; *14: *I. perrierii*.

**Figure 3:**
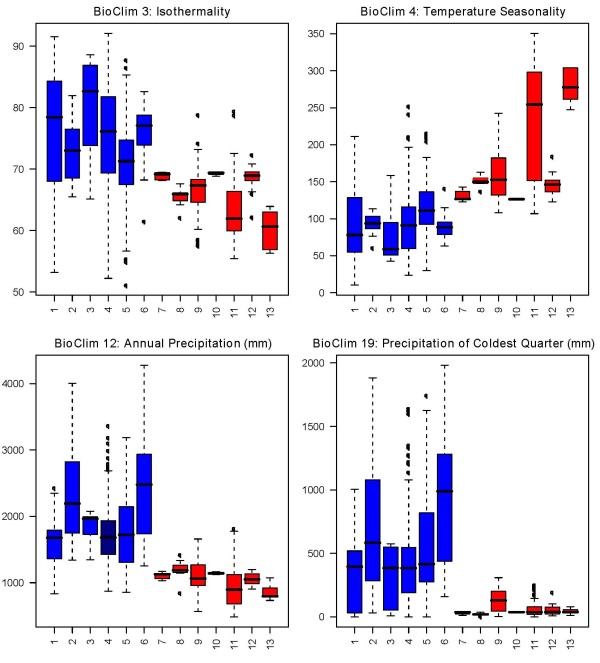
**Climatic variation in species of *Monodora***. Only species sampled in the molecular phylogeny are represented. Blue: West/Central African species; Red: East African species. West/Central African species 1: *Monodora angolensis *2: *M. crispata*, 3: *M. laurentii*, 4: *M. myristica*, 5: *M. tenuifolia*, 6: *M. undulate*. East African species: 7: *M. carolinae*, 8: *M. globiflora*, 9: *M. grandidieri*, 10: *M. hastipetala*, 11: *M. junodii*, 12: *M. minor*, 13: *M. stenopetala*.

For most sampled species there were more than ten unique locality data points, except for *Isolona capuronii *(1), *I. pleurocarpa *(7), *Monodora carolinae *(7), *M. globiflora *(6), *M. hastipetala *(4) and *M. stenopetala *(6), all being rare or localized species. Analysis of the variation of each bioclim variable between species underlines important ecological characteristics within and between species of each genus. For *Isolona*, differences between East and West/Central species were few (Figure 2) and included lower Isothermality (BC3), higher temperature seasonality (BC4) and lower annual precipitation (BC12) for the East African species. In contrast, East African species of *Monodora *showed differences for several variables when compared to the West/Central African ones. For example, they are subject to lower isothermality (BC3), higher temperature seasonality (BC4) and lower annual precipitation (BC12) as well as other related variables such as BC13, BC14, BC16, BC17 and BC19 (Figure 3). Montane restricted taxa (> 1100 m) such as *Isolona congolana*, *I. linearis *and *Monodora globiflora *are exposed to lower mean temperatures (BC8, BC9, BC10 and BC 11, see additional files 1, 2, 3, 4, 5, 6, 7 and 8 for all the variables).

### Sister species comparisons: principal component analysis

**Figure 4:**
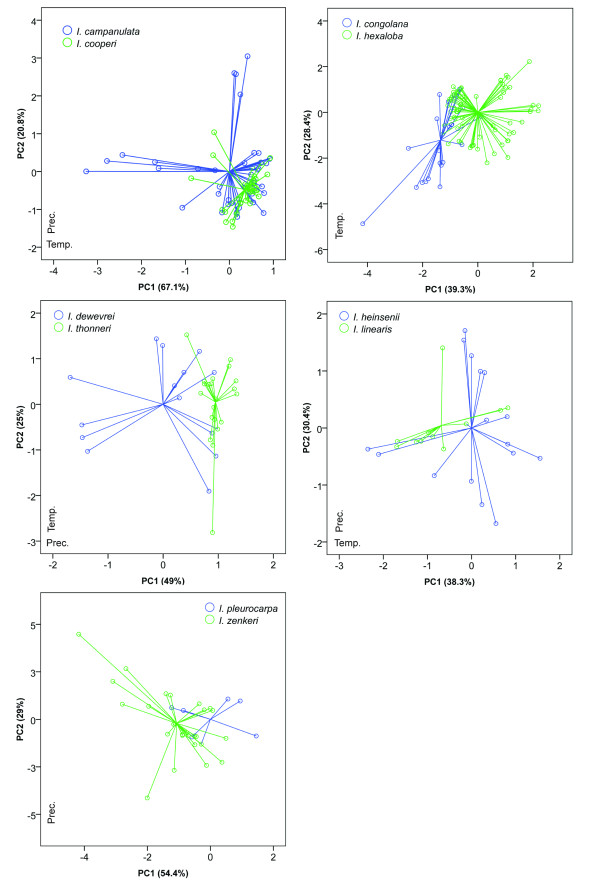
**Climatic comparison between sister species in *Isolona***. Principal component analysis (PCA) between five species pairs. Prec. Precipitation dominant axis identified by the component matrix; Temp.: Temperature dominant axis identified by the component matrix.

**Figure 5:**
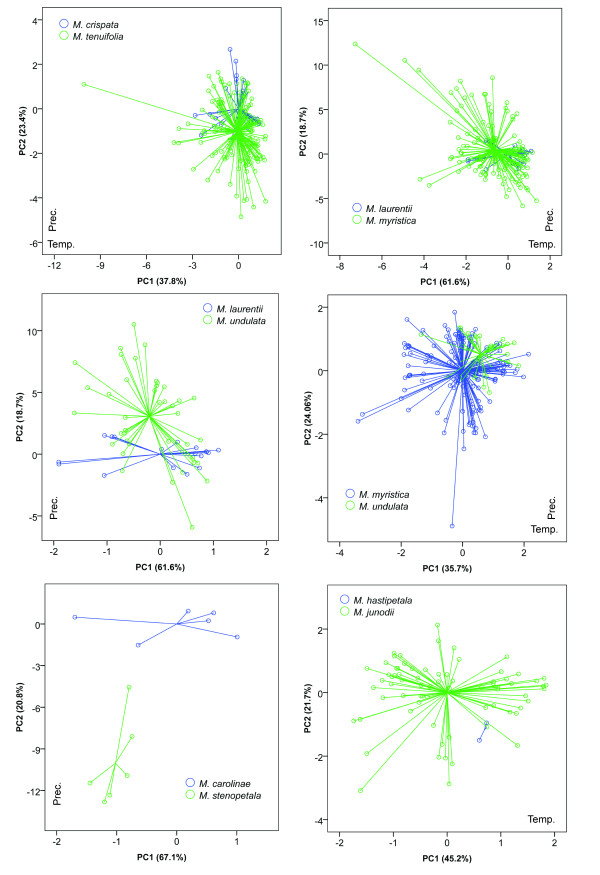
**Climatic comparison between sister species in *Monodora***. Principal component analysis (PCA) between six species pair. Prec. Precipitation dominant axis identified by the component matrix; Temp.: Temperature dominant axis identified by the component matrix.

Environmental niches were compared between sister species using Principal Component Analysis (PCA) on all 19 bioclim variables and significance between principal components were tested under Mann-Whitney U test. Together, the first and second components explained between 59% and 87% of the variation among the 19 bioclim variables. The directionality of the loadings for components 1 and 2 were quite variable, (Figures 4 and 5) but in general, one axis was related to variation in precipitation while the other was related to temperature variation.

PCA indicated environmental overlap between most pairs of sister species. Three main patterns were observed: 1) Species with narrower distributions were included within the environmental variation of a more widely distributed species (for example *Monodora laurentii *within *M. myristica*; *M. hastipetala *within *M. junodii*; *Isolona cooperi *within *I. campanulata*; 2) species that overlapped for part of their variation (for example *M. laurentii */*M. undulata*; *Isolona congolana */*I. hexaloba*); 3) species with clearly separated ecological space, which was found in just one species pair: *Monodora carolinae */*M. stenopetala*. For most comparisons one to two PC axes were not significantly different between species pairs (Table 1). Only the species pairs *M. myristica */*M. undulata *and *I. congolana */*I. hexaloba *were significantly different on all axes tested.

### Niche modeling

Ecological niche modeling was undertaken on all sampled species using Maxent [35]. The area under the curve (AUC) values for all species models ranged from 0.9194 to 0.998 (21 with an AUC higher than 0.95) indicating reliable model performance (see additional file 9). In four models (all of them for *Monodora *species) no standard deviation (SD) was calculated, even though the respective AUCs were high, because the number of total samples was too low (for species with occurrence points less than 8 only one training point can be used from which the SD cannot be estimated). Although several species in this study exhibited low sample sizes, the resulting AUCs indicate that meaningful models have been produced (see additional file 9). The Maxent software has been documented to produce models with good predictive performance from small numbers of sample localities [36-38].

The environmental variable that had the highest contribution to the prediction of each species when training the models (highest training gain) is reported in Table S1. Response curves and variable importance were examined; however no general trends or consistent suite of variables were identified as the important factors for species' distributions.

### Sister species comparisons: Niche similarity tests

**Figure 6:**
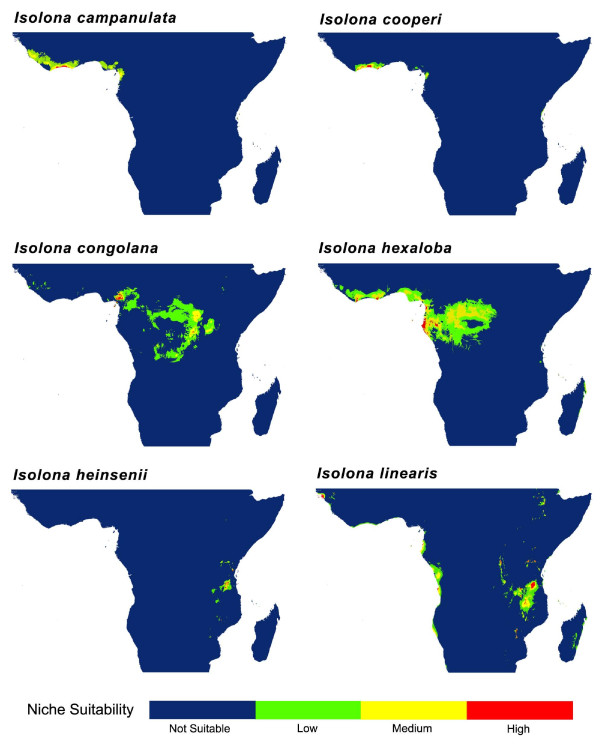
**Potential species distribution between three sister species in *Isolona***. Species distribution generated using MaXent on the 19 bioclim variables.

**Figure 7:**
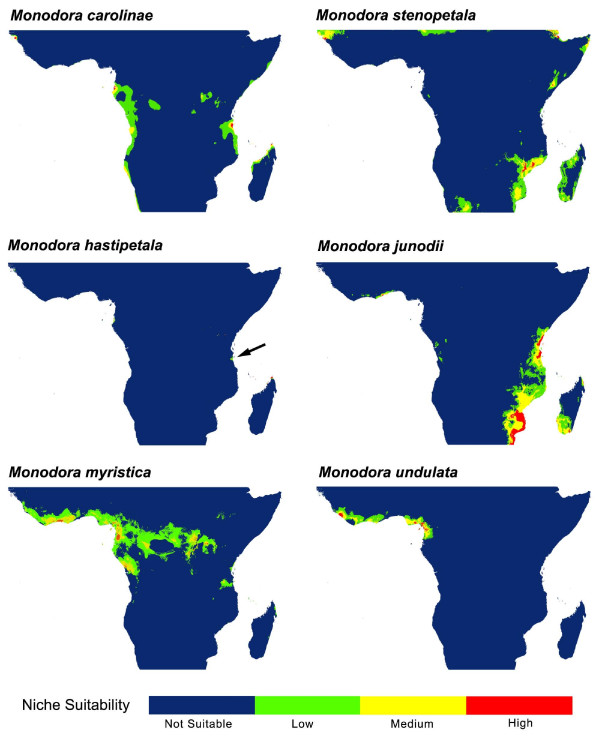
**Potential species distribution between three sister species in *Monodora***. Species distributions generated using MaXent on the 19 bioclim variables. Arrow indicates a small potential distribution.

Potential distribution generated under ENM using Maxent between selected sister species are presented in Figures 6 and 7 and for all species in additional files 10, 11, 12 and 13. Niche identity tests indicated significant ecological differentiation between all sister species (Table 1) demonstrating that ecological niches between species pairs are not identical. In contrast, background tests indicated that in 21 of the 44 tests undertaken (2 (D and I statistics) × 2 (two way tests) × 11) the niches of sister species are more similar than expected by chance alone (Table 1). In 17 cases, the results were not significantly different than the null distribution. Finally, 6 comparisons showed less niche similarities than expected, namely two tests for the species pair *Monodora hastipetala */*M. junodii*, and all four tests for *Isolona heinsenii */*I. linearis*.

### Phylogenetic signal of climatic variables

Two statistics were used to assess the phylogenetic signal of each bioclim variable: the quantitative convergence index (QVI) of Ackerly et al. [39] and the *K *of Blomberg et al. [40]. Tests were undertaken on each genus independently and then on the clade as a whole (Table 2). The QVI varied from 0.34 to 0.79 in *Isolona*, 0.23 to 0.99 in *Monodora *and 0.46 to 0.85 in the whole clade (Table 2). The randomization tests on 1000 posterior trees indicated that 9/19 variables in *Isolona*, 10/19 variables in *Monodora *and 14/19 variables in the whole clade were significantly *smaller *than expected by chance (mean QVI in less than the 99^th ^percentile) indicating strong correlation of those variables with the phylogeny. Interestingly, variables identified with significant phylogenetic signal were not always the same between both genera. In *Isolona*, temperature related variables showed more signal than precipitation ones (6/11 vs. 3/8), whereas in *Monodora *precipitation variables showed more signal (4/11 vs. 6/8). For example, BC17 (Precipitation of Driest Quarter) presented the highest phylogenetic signal (i.e. the lowest values of QVI, Table 2) within *Monodora *whereas BC4 (Temperature Seasonality) showed the highest phylogenetic signal in *Isolona*.

**Table 2:** Test of phylogenetic signal of the 19 bioclim variables using two methods: the quantitative convergence index (QVI) and Blomberg et al. *K*.

	QVI						*K*		
	***Isolona***		***Monodora***		**clade**		***Isolona***	***Monodora***	**clade**

**Bioclim variable**	**Mean QVI**	**Mean QVI random**	**Mean QVI**	**Mean QVI random**	**Mean QVI**	**Mean QVI random**			

bc1	0.53	0.80	0.73	0.83	**0.68**	0.85	**1.03**	0.59	0.49
bc2	0.73	0.82	0.99	0.87	0.85	0.86	0.80	0.33	0.30
bc3	**0.48**	0.79	**0.45**	0.82	**0.55**	0.82	**1.16**	**1.18**	**0.72**
bc4	**0.34**	0.78	**0.50**	0.83	**0.49**	0.82	**1.53**	0.64	**0.60**
bc5	0.60	0.81	0.92	0.87	0.78	0.85	0.89	0.44	0.35
bc6	**0.53**	0.82	0.72	0.90	**0.67**	0.85	**1.14**	0.75	**0.61**
bc7	**0.54**	0.80	0.81	0.84	0.74	0.82	**1.32**	0.40	0.35
bc8	0.72	0.84	0.77	0.79	0.80	0.85	0.79	0.49	0.38
bc9	**0.53**	0.83	**0.52**	0.87	**0.63**	0.86	**1.03**	0.77	**0.59**
bc10	0.68	0.81	0.90	0.80	0.81	0.83	0.81	0.51	0.39
bc11	**0.45**	0.81	**0.43**	0.85	**0.55**	0.84	**1.22**	0.79	**0.64**
bc12	0.62	0.83	**0.23**	0.78	**0.46**	0.83	0.90	**1.44**	**0.86**
bc13	**0.50**	0.80	0.66	0.90	**0.56**	0.82	**1.17**	0.76	**0.66**
bc14	0.69	0.83	**0.24**	0.81	**0.52**	0.83	0.75	**1.25**	**0.70**
bc15	0.64	0.79	**0.35**	0.83	**0.54**	0.83	0.70	**1.45**	**0.76**
bc16	**0.56**	0.81	**0.55**	0.86	**0.56**	0.82	**1.06**	0.77	**0.66**
bc17	0.79	0.86	**0.17**	0.78	**0.46**	0.82	0.68	**1.91**	**0.77**
bc18	**0.35**	0.85	0.94	0.84	**0.52**	0.88	**1.22**	0.63	**0.84**
bc19	0.77	0.82	**0.23**	0.79	**0.53**	0.82	0.90	**1.07**	**0.66**

Mean QVI is the average of the observed QVI across 1000 posterior trees

Mean QVI random is the expected QVI after 1000 randomizations.

Bold values indicate variables that are significantly different than expected from random (P-value < 0.05).

The *K *statistic was higher than expected under a random evolutionary process (K > 1) and showed phylogenetic signal (after a randomization test) for 10 variables in *Isolona *and just 6 in *Monodora *(Table 2). These variables were the same as identified under the QVI for each genus. Only in *Isolona *was one variable (BC1) detected as having a significant signal not found when using the QVI (Table 2). When *K *was calculated for the clade as a whole all of the variables were lower than 1, indicating a shortage of phylogenetic dependence under a random evolutionary model. However, 13/19 showed significant values when compared to the random distribution of the variables on the tree. These were generally the same as those identified on the whole clade using the QVI. This would suggest that our data is robust to phylogenetic uncertainty because the mean QVI and its significance were based on 1000 posterior trees (see Methods).

### Phylogenetic signal of ecological divergence

**Figure 8:**
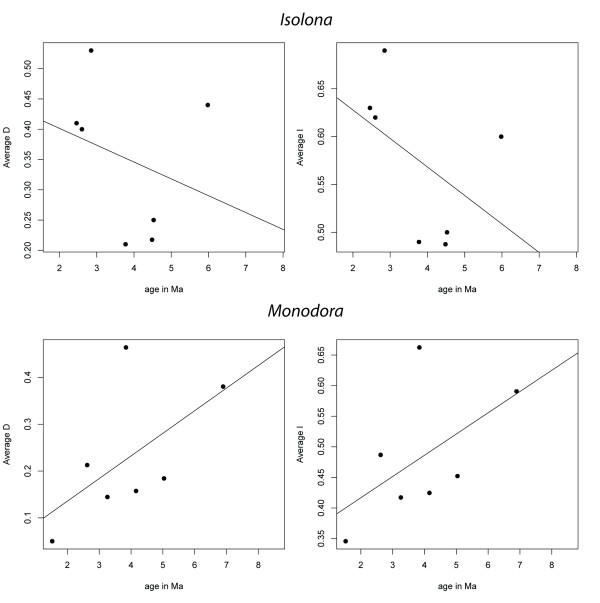
**Phylogenetic signal of niche similarity for both genera using D or I in function of time**. Line is the fitted regression.

An adaptation of the age-range correlation method following [26] was used to test if there was a phylogenetic signal in ecological divergence using the niche similarity indices I and D [26]. The slope of the regression in *Isolona *was negative supporting the idea of an increase in niche differences with phylogenetic distance. In contrast, *Monodora *had a positive slope of the regression (Figure 8) which indicates that niche similarity among the species increases through time. However, neither of these analyses were significantly different than the null hypothesis (Table 3) indicating the absence of a significant phylogenetic signal of the ecological niche.

**Table 3:** Significance results of the Age-Range Correlation (ARC) analyses using randomization tests under Monte Carlo resampling.

	I				D			
	Intercept	f(greater)^1^	slope	f(greater)^1^	Intercept	f(greater)^1^	slope	f(greater)^1^
*Isolona*	0.68	0.118 (NS)	0.736	0.528 (NS)	0.45	0.316 (NS)	0.637	0.726 (NS)
*Monodora*	0.347	0.807 (NS)	0.145	0.29 (NS)	0.039	0.79 (NS)	0.166	0.332 (NS)

^1^The proportion of Monte Carlo replicates with greater slopes or intercept than the observed value.

## Discussion

### Ecological dimensions of speciation

This study is to our knowledge the first one explicitly using ecological niche models and phylogeny to understand speciation in a clade of African rain forest trees. Our results indicate little geographical overlap between sister species (7/11 comparison with less than 30% overlap, Table 1). Thus, most species pairs occur in allopatry, a result consistent with the geographical isolation hypothesis, although this would also be expected under the ecological gradient hypothesis. In one case the overlap was relatively large, for example between *M. junodii *and *M. hastipelata *(~ 90%). However, this might be an artifact in the way the overlap statistic was calculated (see Methods) as both these species never grow in complete sympatry (i.e. next to each other, pers. obs.). *Monodora hastipelata *is a local endemic to a small area in the Matumbi Hills in Tanzania [41] while *M. junodii *has an extensive distribution across East Africa [28].

Most species pairs show visual overlap in ecological space (Figures 4 and 5) and are never completely separated by PCA analysis (expect for the *M. carolinae *and *M. stenopetala *pair, see below). In contrast, the niche similarity tests [26] based on the ecological niche models demonstrated that all species pair had highly significantly different niches (Table 1). This test, however, is known to be very strict, and rejection of similarity can occur based on very small niche differences, especially for allopatric species [42]. At the scale studied here (distribution of species at the continental level) the probability of two species having completely *identical *niches is very low [18] and these tests might be too stringent, something already noted in other studies (e.g. [43]). The background tests appear better suited for this type of analyses and is generally used when species occur in allopatry [42]. Just under half of the tests undertaken (48%, Table 1) supported the hypothesis of niche similarity between sister species whereas 13% (6/44) of the tests supported a significant difference. However, four of these latter tests were found for the species pair *Monodora junodii/M. hastipetala*, and could be linked to the few data points associated with *M. hastipetala *(see Methods). In several cases (17/44) a non-significant (NS) result was found, which is suggestive of a lack of power to detect niche differentiation/similarity, either linked to a low sample size or to the distribution of the habitat [26]. NS results were found either between widely distributed species with a fair number of collections (e.g. *I. hexaloba *and *M. myristica*), suggesting habitat heterogeneity as a source of lack of power, or between species with a small number of collections (*M. stenopetala */*M. carolinae*; *I. thonneri */*I. dewevrei*).

Finally, we also tested each bioclim variable for phylogenetic signal, i.e. the statistical nonindependence among species of the variables given their phylogenetic relationships. Direct interpretation in terms of evolution of significant phylogenetic signal of traits is difficult and should be done with caution [44,45]. These tests yielded different results depending on the level of the analysis (genus versus clade). Nevertheless, both statistics identified several bioclim variables within each genus and for the clade as containing significant phylogenetic information (Table 2). Such a result was already suggested for *Monodora *[31] but not for *Isolona *or the clade as a whole. However, it should be noted that for the genus tests the power of the randomization approach to detect significance of the K statistic might be slightly low for 14-16 tip phylogenies [40]. Here, we do not attempt to draw strong conclusions about the rate of evolutionary change or the pattern of the evolutionary process linked to these variables. We simply underline that many variables are not randomly placed on the phylogeny and that they deviate from a random evolutionary process as generated under the Brownian motion model (as interpreted by the K statistic). This is what would be expected under niche conservatism and the geographical speciation model, although exactly how they have influenced its diversification would require more in depth model fitting analyses [44,46]. In addition, we failed to identify any phylogenetic signal of ecological niche overlap (Figure 7) [26]. Absence of phylogenetic signal can be the result of a mixture in the speciation pattern [47], or it may be directly related to the quality of the data. In *Monodora*, the latter would appear to be the case as phylogenetic resolution within the West species is low, and specimen locality data from two sister species pair in East Africa are few, both being sources of error.

The analyses at several levels using different approaches provide a mixed signal on the role of ecology in speciation. Although sister species within this clade do not have identical niches, which is to be expected [18], they are in several cases significantly more similar between each other than by chance alone (Table 1). Overall however, based on PCA, ecological niche modeling and phylogenetic signal analyses, our results do provide some support to the idea that in terms of diversification, ecological speciation as viewed through climate has not played a major role in the evolution of *Isolona *and *Monodora *species. This contrasts with several recent publications where significant ecological divergence was generally demonstrated for northern hemisphere plant sister species such as in *Lonicera *[48], *Cyclamen *[24], five Andean *Solanum *species [25] and in most clades of the South American genus *Hordeum *[43]. They all concluded that ecological speciation was an important factor of diversification within these genera. Moreover, in the mainly African distributed cucurbit genus *Coccinia*, frequent biome shifts were inferred during a period of 6 Ma between forest, woodland and semi arid habitats, implying an important role of ecological diversification [49]. Finally, the spread of arid environments in Africa during the Neogene was suggested to be an important driver of diversification of some partially TRF genera such as *Acridocarpus *[21]. Adaptation to alternative (more arid) environments appears extremely limited within *Isolona *and *Monodora *mainly because of the strong evolutionary constraint applied by the precipitation variables (Table 2). A strong correlation between rainfall and species distribution was also found within Neotropical Annonaceae in general [50].

The only species pair for which speciation might have relied on ecology is found in the East African species *M. carolinae *et *M. stenopetala *(Figure 5), the former occurring in moist semi-deciduous coastal forests of southern Tanzania and Mozambique while the latter is found in dense thickets and woodlands of Malawi (Figure 7). However, the background test indicated that *M. stenopetala *had a significantly more similar niche when compared to the environmental background of *M. carolinae *(in the other direction, i.e. the similarity of *M. carolinae *using the background of *M. stenopetala*, was non-significant, and thus inconclusive). This result is quite intriguing given the important ecological differences identified using PCA between these two species, and could be an artifact due to the low sample size of both species (7 for *M. carolinae*; 6 for *M. stenopetala*). It remains unclear exactly how many samples are necessary to produce a robust ecological niche model, although some authors have suggested more than 10 [23,27] or even over 100 [see [51]]. Also, these results could be due to the established background that was used for the tests, here defined as all grid cells within 20 km of known occurrence localities. The background tests are known to be sensitive to the definition of species 'background' and therefore a finer or coarser background region could yield different results [25].

### Temporal dimensions of speciation

The estimated mean ages of the origin of species in both genera inferred by Couvreur et al. [29] (Figure 1, Table 1) are dated to before or at the beginning of the Pleistocene (9/11 speciation events are older than 2.4 Ma, Table 1). This would suggest that the Pleistocene climatic fluctuations had little effect on species diversity within these genera. This result is intriguing because the geographical isolation hypothesis was thought to be especially important during the Pleistocene because of the numerous successive rain forest expansion and fragmentation [52-54]. Thus, although ecology plays a little role in the speciation processes of these genera in Africa, the timing of these events pre dates a period of intensive climatic variation and hence potential isolation events. Rather, most speciation events occurred in the Late Miocene and during the Pliocene (between 6-2.4 Ma). It is important to note that these conclusions are based on the mean age for each node and that the 95% confidence intervals largely overlap with the Pleistocene (see Table 1), and thus should be treated with caution. However, these estimates are in line with numerous other studies that have identified pre-Pleistocene diversification in African TRFs, such as in *Afromomum *[55], an estimated 60% of *Begonia *species [56] as well as in several animal clades such as African birds [57] and African clawed frogs species [58]. This was also recorded for the genus *Coccinia *[49], although this genus is not restricted to TRF. The Late Pliocene corresponds to a period of renewed rain forest re-expansion which was preceded by a fairly long period of aridification and savanna expansion during the Late Miocene [52]. In contrast, a recent temporal analysis of the herbaceous and mainly montane genus *Impatiens *suggested an important role of Pleistocene refuges on the diversification of the genus [59]. Most of the studies that have detected (some) Pleistocene diversification focused on herbaceous plant clades (e.g. *Impatiens *and *Begonia *[9,56,59]) which are known to have faster rates of molecular evolution when compared to woody taxa (e.g. trees) [60] and or dispersal abilities. Intraspecific analyses of genetic diversity (phylogeography) of widespread African tree (woody) species (including an African Annonaceae species *Greenwayodendron suaveolens *[61]) suggest that Pleistocene refuges did have some effect but mainly at the infra specific genetic structure level only [61,62]. The small role of the Pleistocene in generating species was also suggested to be the case in *Afromomum *[55]. Even though *Afromomum *species are herbs, individuals can live up to 10 years which could imply lower molecular evolution [55] when compared to other herbaceous taxa. The climatic variations of the past 2.5 Ma might have been too quick to allow allopatric speciation with little ecological divergence to operate, especially on organisms with lower rates of molecular evolution such as trees, something that is supported by our data as well as in other studies [49]. Thus, the late Miocene and Pliocene epochs appear to have played a significant role in diversification of African TRF trees possibly related to the longer phases of unfavorable climatic conditions and isolation of populations allowing proper genetic isolation between them.

### Conclusion

Although it is generally argued that ecology is never truly absent from speciation [63], it is important to understand to what extent it can influence species production over time. Our results imply that adaptation to climatic differences between sister species have not been a major driver of speciation in trees of African TRFs, which is consistent with the geographical speciation model of TRF diversification. Such a result would seem to contrast with other patterns detected in non-TRF plants. In the absence of other evidence, speciation in the studied genera could possibly be the result of intricate pollination mechanisms. Indeed, both genera present strikingly different flower morphologies (Figure 1), and intra generic variation is also important [28]. Unfortunately, to date, little information is available on African Annonaceae pollination biology [28,64], something that should be further explored.

## Methods

### Divergence dates

For this analysis we used the chronogram of Couvreur et al. [29] which included 14 out of 20 species of *Isolona *and 13 out of 14 species of *Monodora*. The analysis was based on five chloroplast markers and the tree was dated under a relaxed clock model with uncorrelated rates across lineages [see 29 for details]. A secondary calibration point was used, with the crown node of *Isolona *and *Monodora *set to 14.9 Ma (95% highest posterior density (HPD) 9.4-21). A similar age (14.4 (95% HPD 10.2-18.7) for this node was also found with a larger sampling of Annonaceae genera and with an updated fossil calibration hypothesis [65].

### Locality data and geographical distribution

Locality data were compiled from Couvreur [28] and represented over 1500 georeferenced herbarium specimens (see additional files 14, 15, 16, 17 and 18 for distribution maps of all species included in this study). All localities were imported into ArcGIS v. 9.3 [66] and projected to an Albers projection with a World Geodetic System 1984 datum. Uncertain georeferenced specimens as well as specimens from identical localities for each species were deleted from the database prior to analyses, leaving a total of 335 unique occurrence localities for *Isolona *and 737 for *Monodora *(Table S1).

An estimate of the known geographic range for each species was produced in ArcGIS v 9.3 using a "buffer" approach. This method creates a buffer radius around each collection point for each species. Overlapping buffers for each species pair are then fused and the range overlap is calculated. Several other approaches can be used such as the "quadrat" [e.g. [67]]: the distribution of species are broken down into "pixels" (for example one-degree grid cells) and overlap is calculated based on how many pixels each species have in common; or via a "minimum convex polygon" [68]: the distribution of the species is represented by a polygon which contains all data points with no angle larger than 180 degrees. However, both these approaches will be biased in a certain way: in the former case two data points of two different species could be very close together but be considered as not overlapping as the points are in two opposing corners of different pixels; in the later it has been shown to overestimate distribution ranges as large areas are included even though there are no collections [68]. The buffer approach is suitable because the method is based on the data point itself, however, the size of the buffer can produce a bias. Different buffer sizes were here investigated (2, 10 and 20 km), and we chose the results of the 20 km buffer size as it best captures the patchy nature of these species' distributions at the spatial scale used in this study. All buffers were then merged and the area within buffers was calculated for each individual species. Basic overlay functions were used to estimate the percentage of known geographic range overlap for each sister species pair, where the amount of buffered area overlapping between two species was divided by the total buffered area for the species with the smaller range following [25].

Using the 19 bioclim variables (Table 4) from http://www.worldclim.org at 30 arc seconds resolution [69], a set of climatic measurements that summarize temperature and precipitation dimensions of the environment, values were extracted for each unique specimen locality using the 'Extract Values to Points' tool in the Spatial Analyst extension of ArcGIS v. 9.3. These values were then used to visualize climatic variability for each species.

**Table 4:** Environmental (bioclim) variables used to construct ecological niche models in Maxent.

Abbreviation	Description
BIO1	Annual mean temperature
BIO2	Mean diurnal range (mean of monthly (max temp - min temp))
BIO3	Isothermality (BIO2/BIO7) (* 100)
BIO4	Temperature seasonality (standard deviation *100)
BIO5	Max temperature of warmest month
BIO6	Min temperature of coldest month
BIO7	Temperature annual range (BIO5-BIO6)
BIO8	Mean temperature of wettest quarter
BIO9	Mean temperature of driest quarter
BIO10	Mean temperature of warmest quarter
BIO11	Mean temperature of coldest quarter
BIO12	Annual precipitation
BIO13	Precipitation of wettest month
BIO14	Precipitation of driest month
BIO15	Precipitation seasonality (coefficient of variation)
BIO16	Precipitation of wettest quarter
BIO17	Precipitation of driest quarter
BIO18	Precipitation of warmest quarter
BIO19	Precipitation of coldest quarter
ALT	Altitude

### Sister species comparisons: PCA

Environmental niches were compared between sister species using Principal Component Analysis (PCA) on all 19 bioclim variables. Statistical tests between groups have generally relied on AMOVA or MANOVA methods either between the principal components of the PCA [27,48] or on the climatic variables directly [25]. However, these tests can only be undertaken if the underlining assumptions of ANOVA are met: normal distribution of the data and homogeneity of the variance. In our case, both the bioclim variables and the PCA components violated those assumptions (One-Sample Kolmogorov-Smirnov test rejected the normality of the data in all cases and Levene's test rejected the equality of error variances in all cases (data not shown)). Thus, statistical differences between sister species were assessed using the non parametric Mann-Whitney U test by comparing the principal components (PC1, PC2, PC3) [27,48].

### Niche modeling

Ecological niche modeling was used in order to summarize the climatic tolerances of the sampled species, except for *M. hastipetala *(see below). Ecological niche models were generated using the maximum entropy method, Maxent version 3.3 [35]. This is a presence-only method demonstrated to perform well when compared to similar approaches [37,70,71]. Maxent generates a continuous probability distribution of habitat suitability for each input species. The software finds the distribution that is closest to uniform, or of maximum entropy, within the study area, and it does so subject to the constraints imposed by variations in the environmental variables at the species' occurrence localities [35].

The study area used for niche analyses included Africa and Madagascar and was confined to the known northern and southern extent of *Isolona *and *Monodora *(below 12°51'N and above 28°7'S); latitudinal boundaries which roughly coincide with limits of the suitable land cover types for these species. The 19 bioclim layers and an elevation layer (Table 3), downloaded from the Worldclim data set, were used as environmental variables in the models [69]. For each species, a total of 100 replicates were run with random seed, which creates a different random data partition (25% test, 75% training) for each run. To choose presence data for each replicate, bootstrapping allowing sampling with replacement was used. For further analyses, the averaged Maxent output from these 100 models was used. All models were run under auto-features in logistic format [35], using a maximum of 500 interactions and regularization multiplier of 1.0. The importance of individual environmental variables in explaining the distribution of each species modeled was determined by running jackknife tests within the Maxent interface [35]. The area under the curve (AUC) of the receiver operating characteristic (ROC) plot was employed to evaluate model performance [72]. AUC is a threshold-independent measure that quantifies the ability of a model to distinguish presence data from background data as compared to a random prediction. AUC values range from 0 to 1, with 0.5 denoting a model that is performing no better than random. Higher AUC values indicate better performing models and models with an AUC value over 0.7 are considered useful [73] but see [74].

### Sister species comparisons: niche similarity tests

For all sister species pairs, we compared the Maxent outputs using the software ENMtools [42] following the methods described in [26,42]. For one species (*Monodora hasitpetala*), niche models we unable to be generated in Maxent due to the low number of unique occurrence localities (4). Therefore, the known geographic range was used for niche similarity tests, rather than the Maxent output. The software quantifies niche similarity using two metrics: *D *[75], and *I*, a measure derived from Hellinger distance. These metrics are calculated by comparing the estimated niche suitability values from individual pixels in Maxent model outputs, where those outputs have first been normalized such that all predicted suitability values in the geographic space sum to 1 [26]. Although both similarity measures are calculated in a similar manner, they differ in how the interpretation of the niche suitability values. The results for both measures range from 0 (no niche overlap) to 1 (identical niches). In ENMtools, niche overlap was calculated for each of the sister species pairs. Additionally, two randomization tests were run in ENMtools to evaluate niche similarity and conservatism between sister species only: niche identity and background similarity tests [26].

The niche identity test compares niche models generated with actual occurrence localities to pseudoreplicate models generated with points randomly selected from a pool of actual occurrence localities to determine if species pairs have equivalent niches. For the identity tests, 100 pseudoreplicates were created from the pooled localities for each pair of sister species and *D *and *I *values were calculated for each of the pseudoreplicate models. The distribution of these similarity values was then compared to the *D *and *I *values calculated from the actual niche models for that species pair in the niche overlap test. This method tests the null hypothesis that the two species have equivalent ecological niches and is expected to be met only if both species tolerate exactly the same environmental conditions and have an equivalent set of environmental condition available to them [26].

The background similarity test compares differences in the environmental background of species pairs (as opposed to the actual occurrence localities) to determine if the two species are more or less similar than expected by chance. For each species pair, the niche model for the focal species is compared to a series of pseudoreplicate models generated by randomly sampling the 'background' (geographic range) of its sister species [26]. In the context of the similarity test, the known geographic range previously calculated for each of the study species was defined as its background, and 100 pseudoreplicates were created for each species pair tested. *D *and *I *values were calculated for each pseudoreplicate model and the distribution of these values was compared to the niche overlap values calculated for the actual data. This method tests the null hypothesis that calculated niche overlap between two species is explained by differences in their environmental background. The null hypothesis is rejected if the calculated niche overlap falls outside the 95% confidence interval for the distribution of pseudoreplicate model values.

### Phylogenetic signal of bioclim variables

The phylogenetic signal (we prefer the term "phylogenetic signal" over "phylogenetic conservatism" as suggested by [20]) for each bioclim variable was tested on both genera independently using two methods specifically designed for continuous characters: the quantitative convergence index (QVI) of Ackerly and Donoghue [39] and the *K *of Blomberg et al. [40]. The QVI represents the inverse of the retention index for continuous characters. When QVI = 0 similar species for a trait are sister taxa, and when QVI = 1 similar species for that trait are not closely related. The calculation of the QVI was undertaken on 1000 randomly chosen posterior trees and with 1000 randomizations of the tree tips using the software program CACTUS 1.13 [76]. This approach allows to take phylogenetic uncertainty into account when calculating the QVI.

*K *is used to quantify the "amount of phylogenetic signal relative to the amount expected for a character undergoing Brownian motion evolution along the specified topology and branch lengths" [[40], page 730]. This statistic differs from the previous one as it tests the degree of resemblance of the (continuous) variables between sister species under an explicit null evolutionary model: the Brownian motion model [40]. The statistic varies from 0 to infinity, with *K *< 1 indicating low phylogenetic dependence of the variable and K > 1 indicating high phylogenetic signal of the variable. When *K *= 1, the variable exhibits the phylogenetic signal expected under the Brownian motion model (e.g. the null model). *K *was estimated for each of the 19 bioclim variables using the *multiPhylosignal *command in the *picante *(ver. 1.3) [77] R package and its significance was assessed by 999 randomizations.

### Phylogenetic signal of niche differentiation

We also tested for phylogenetic signal of niche differentiation by using an adaptation of the age-range correlation method of [47] in which niche similarity indices I and D are viewed in function of time following [26]. I and D values were estimated between all species pairs for each genus. We used the R package *phyloclim *ver. 0.8.1 [78] to generate the correlation graphs. Phylogenetic signal was tested by Monte Carlo simulations to randomize the I and D indices of each species in order to estimate of the slope and intercept of the plots under the null hypothesis of no phylogenetic signal [47] as implemented in *phyloclim*. For each genus, a total of 1000 simulations were undertaken.

## Authors' contributions

TLPC conceived and coordinated the study, participated in its design, undertook part of the statistical analyses and drafted the manuscript. HPM participated in the design of the study, performed part of the statistical and all GIS analyses and helped draft the manuscript. JJW participated significantly in the production of the data. LWC participated in the conception of the study. All authors read and approved the final manuscript.

## Supplementary Material

Additional file 1 **Variation of bioclim variables BC1-6 for *Isolona***. Indicates the variation of bioclim variables BC1 to 6 for all sampled species in *Isolona*. West/Central African species: 1: *Isolona congolana; *2: *I. hexaloba; *3: *I. pleurocarpa; *4: *I. zenkeri; *5: *I. campanulata; *6: *I. cooperi; *7: *I. dewevrei; *8: *I. thonneri; *9: *I. cauliflora*. East African species: 10: *I. heinsenii; *11: *I. linearis*. Malagasy species: 12: *I. capuroni; *13: *I. ghesquierei; *14: *I. perrierii*. Click here for file

Additional file 2 **Variation of bioclim variables BC7-12 for *Isolona***. Indicates the variation of bioclim variables BC7 to 12 for all sampled species in *Isolona*. West/Central African species: 1: *Isolona congolana; *2: *I. hexaloba; *3: *I. pleurocarpa; *4: *I. zenkeri; *5: *I. campanulata; *6: *I. cooperi; *7: *I. dewevrei; *8: *I. thonneri; *9: *I. cauliflora*. East African species: 10: *I. heinsenii; *11: *I. linearis*. Malagasy species: 12: *I. capuroni; *13: *I. ghesquierei; *14: *I. perrierii*. Click here for file

Additional file 3 **Variation of bioclim variables BC13-18 for *Isolona***. Indicates the variation of bioclim variables BC7 to 12 for all sampled species in *Isolona*. West/Central African species: 1: *Isolona congolana; *2: *I. hexaloba; *3: *I. pleurocarpa; *4: *I. zenkeri; *5: *I. campanulata; *6: *I. cooperi; *7: *I. dewevrei; *8: *I. thonneri; *9: *I. cauliflora*. East African species: 10: *I. heinsenii; *11: *I. linearis*. Malagasy species: 12: *I. capuroni; *13: *I. ghesquierei; *14: *I. perrierii*. Click here for file

Additional file 4 **Variation of bioclim variable BC19 for *Isolona***. Indicates the variation of bioclim variable BC19 for all sampled species in *Isolona*. West/Central African species: 1: *Isolona congolana; *2: *I. hexaloba; *3: *I. pleurocarpa; *4: *I. zenkeri; *5: *I. campanulata; *6: *I. cooperi; *7: *I. dewevrei; *8: *I. thonneri; *9: *I. cauliflora*. East African species: 10: *I. heinsenii; *11: *I. linearis*. Malagasy species: 12: *I. capuroni; *13: *I. ghesquierei; *14: *I. perrierii*. Click here for file

Additional file 5 **Variation of bioclim variables BC1-6 for *Monodora***. Indicates the variation of bioclim variables BC1 to 6 for all sampled species in *Monodora*. West/Central African species 1: *Monodora angolensis *2: *M. crispata*, 3: *M. laurentii*, 4: *M. myristica*, 5: *M. tenuifolia*, 6: *M. undulata*. East African species: 7: *M. carolinae*, 8: *M. globiflora*, 9: *M. grandidieri*, 10: *M. hastipetala*, 11: *M. junodii*, 12: *M. minor*, 13: *M. stenopetala*. Click here for file

Additional file 6 **Variation of bioclim variables BC7-12 for *Monodora***. Indicates the variation of bioclim variables BC7 to 12 for all sampled species in *Monodora*. West/Central African species 1: *Monodora angolensis *2: *M. crispata*, 3: *M. laurentii*, 4: *M. myristica*, 5: *M. tenuifolia*, 6: *M. undulata*. East African species: 7: *M. carolinae*, 8: *M. globiflora*, 9: *M. grandidieri*, 10: *M. hastipetala*, 11: *M. junodii*, 12: *M. minor*, 13: *M. stenopetala*. Click here for file

Additional file 7 **Variation of bioclim variables BC13-18 for *Monodora***. Indicates the variation of bioclim variables BC7 to 12 for all sampled species in *Monodora*. West/Central African species 1: *Monodora angolensis *2: *M. crispata*, 3: *M. laurentii*, 4: *M. myristica*, 5: *M. tenuifolia*, 6: *M. undulata*. East African species: 7: *M. carolinae*, 8: *M. globiflora*, 9: *M. grandidieri*, 10: *M. hastipetala*, 11: *M. junodii*, 12: *M. minor*, 13: *M. stenopetala*. Click here for file

Additional file 8 **Variation of bioclim variable BC19 for *Monodora***. Indicates the variation of bioclim variable BC19 for all sampled species in *Monodora*. West/Central African species 1: *Monodora angolensis *2: *M. crispata*, 3: *M. laurentii*, 4: *M. myristica*, 5: *M. tenuifolia*, 6: *M. undulata*. East African species: 7: *M. carolinae*, 8: *M. globiflora*, 9: *M. grandidieri*, 10: *M. hastipetala*, 11: *M. junodii*, 12: *M. minor*, 13: *M. stenopetala*. Click here for file

Additional file 9 **Species values**. Indicates the number of unique data points as well as several ENM parameters for each species sampled in the molecular phylogeny used to generate the ENM. Bold values indicate species for which there were fewer than 8 unique data points. Click here for file

Additional file 10 **Potential distribution of *Isolona *species**. Shows the rest of the models generated for *Isolona *species. Click here for file

Additional file 11 **Potential distribution of *Monodora *species**. Shows the rest of the models generated for *Monodora *species. Click here for file

Additional file 12 **Potential distribution of the two last *Monodora *and *Isolona *species**. Shows the rest of the models generated for *Monodora *and *Isolona *species. Click here for file

Additional file 13 **Distribution of species in *Isolona***. Shows the geographical location of all data points for each species used in this study. Click here for file

Additional file 14 **Distribution of species in *Isolona***. Shows the geographical location of all data points for each species used in this study. Click here for file

Additional file 15 **Distribution of species in *Isolona *(continue from sup file 1) and *Monodora***. Shows the geographical location of all data points for each species used in this study. Click here for file

Additional file 16 **Distribution of species in *Monodora *(continue from sup file 2)**. Shows the geographical location of all data points for each species used in this study. Click here for file

Additional file 17 **Distribution of species in *Monodora *(continue from sup file 3)**. Shows the geographical location of all data points for each species used in this study. Click here for file

Additional file 18 **Distribution of species in *Monodora *(continue from sup file 4)**. Shows the geographical location of all data points for each species used in this study. Click here for file
