# Supplementary material for: Little ecological divergence associated with speciation in two African rain forest tree genera
Source: BMC Evol Biol. 2011 Oct 11;11:296. doi: 10.1186/1471-2148-11-296 (PMC3203876; doi:10.1186/1471-2148-11-296)
Supplement: Additional file 1 — Variation of bioclim variables BC1-6 for Isolona. Indicates the variation of bioclim variables BC1 to 6 for all sampled species in Isolona. West/Central African species: 1: Isolona congolana; 2: I. hexaloba; 3: I. pleurocarpa; 4: I. zenkeri; 5: I. campanulata; 6: I. cooperi; 7: I. dewevrei; 8: I. thonneri; 9: I. cauliflora. East African species: 10: I. heinsenii; 11: I. linearis. Malagasy species: 12: I. capuroni; 13: I. ghesquierei; 14: I. perrierii. [file 1471-2148-11-296-S1.PDF]

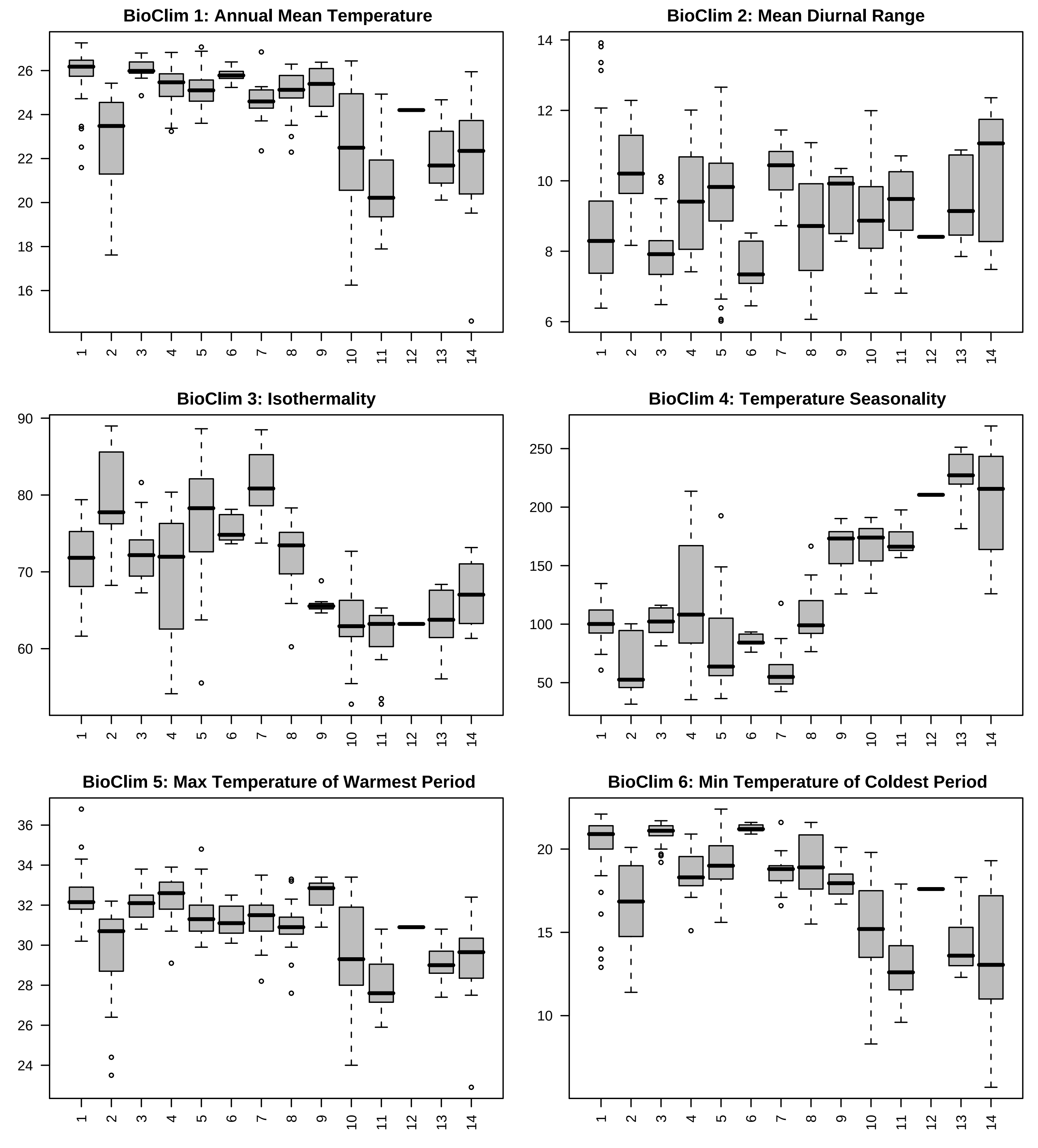
BioClim 1: Annual Mean Temperature
16 18 20 22 24 26
1 2 3 4 5 6 7 8 9 10 11 12 13 14
BioClim 2: Mean Diurnal Range
6 8 10 12 14
1 2 3 4 5 6 7 8 9 10 11 12 13 14
BioClim 3: Isothermality
60 70 80 90
1 2 3 4 5 6 7 8 9 10 11 12 13 14
BioClim 4: Temperature Seasonality
50 100 150 200 250
1 2 3 4 5 6 7 8 9 10 11 12 13 14
BioClim 5: Max Temperature of Warmest Period
24 26 28 30 32 34 36
1 2 3 4 5 6 7 8 9 10 11 12 13 14
BioClim 6: Min Temperature of Coldest Period
10 15 20
1 2 3 4 5 6 7 8 9 10 11 12 13 14
