# Supplementary material for: Little ecological divergence associated with speciation in two African rain forest tree genera
Source: BMC Evol Biol. 2011 Oct 11;11:296. doi: 10.1186/1471-2148-11-296 (PMC3203876; doi:10.1186/1471-2148-11-296)
Supplement: Additional file 2 — Variation of bioclim variables BC7-12 for Isolona. Indicates the variation of bioclim variables BC7 to 12 for all sampled species in Isolona. West/Central African species: 1: Isolona congolana; 2: I. hexaloba; 3: I. pleurocarpa; 4: I. zenkeri; 5: I. campanulata; 6: I. cooperi; 7: I. dewevrei; 8: I. thonneri; 9: I. cauliflora. East African species: 10: I. heinsenii; 11: I. linearis. Malagasy species: 12: I. capuroni; 13: I. ghesquierei; 14: I. perrierii. [file 1471-2148-11-296-S2.PDF]

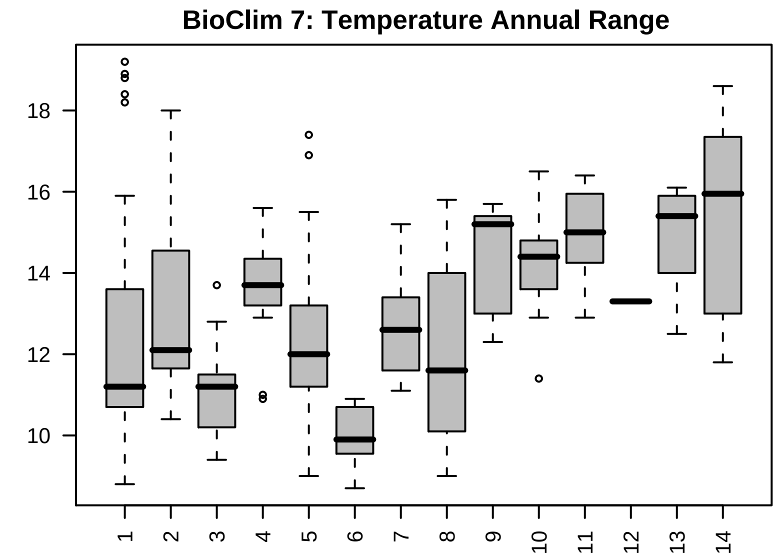
BioClim 7: Temperature Annual Range
10
12
14
16
18
1 2 3 4 5 6 7 8 9 10 11 12 13 14

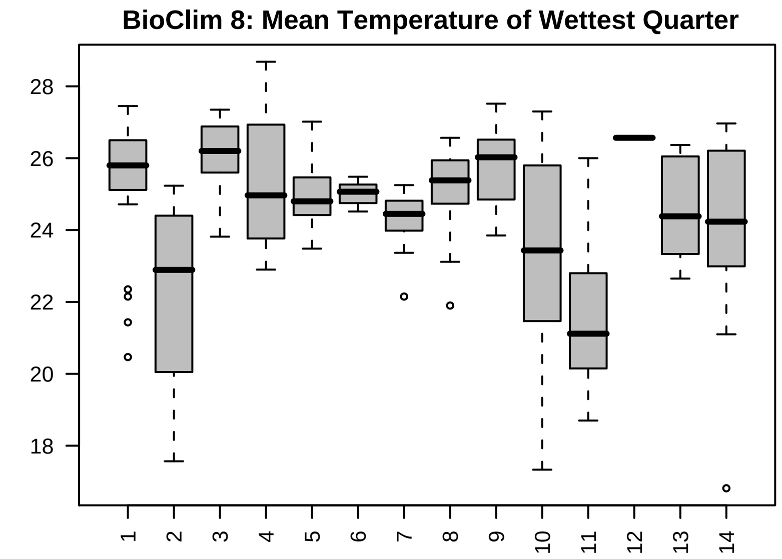
BioClim 8: Mean Temperature of Wettest Quarter
18
20
22
24
26
28
1 2 3 4 5 6 7 8 9 10 11 12 13 14

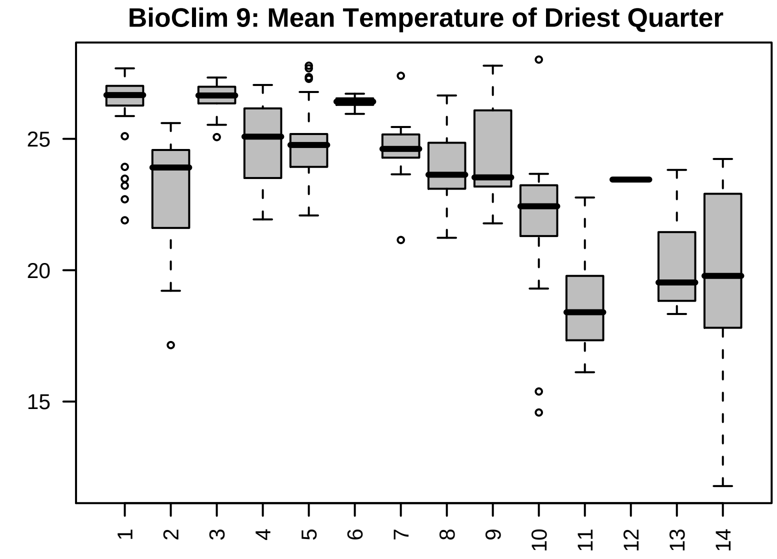
BioClim 9: Mean Temperature of Driest Quarter
15
20
25
1 2 3 4 5 6 7 8 9 10 11 12 13 14

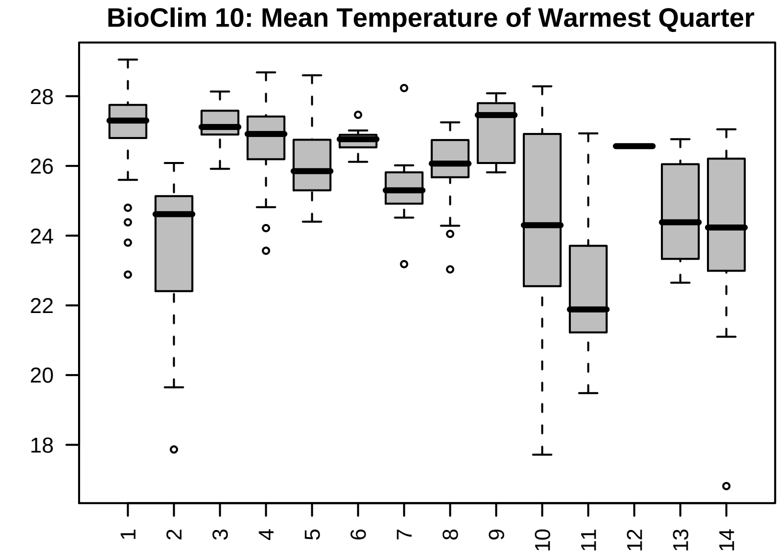
BioClim 10: Mean Temperature of Warmest Quarter
18
20
22
24
26
28
1 2 3 4 5 6 7 8 9 10 11 12 13 14

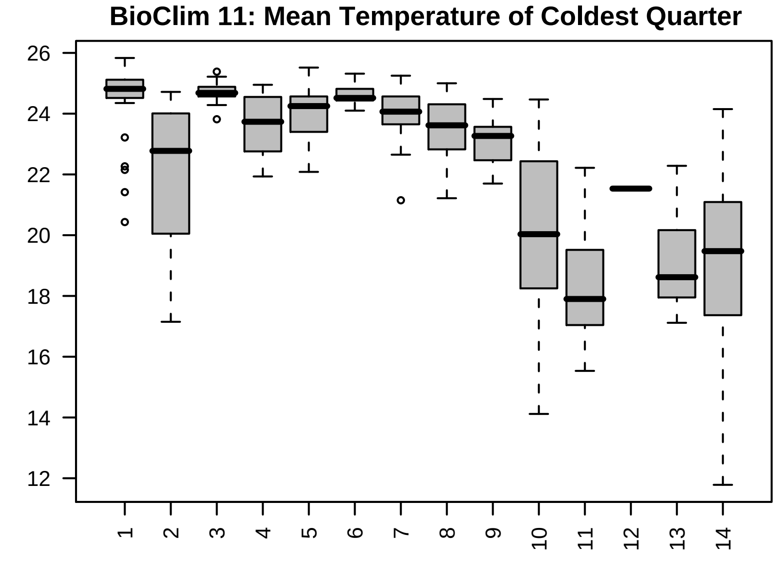
BioClim 11: Mean Temperature of Coldest Quarter
12
14
16
18
20
22
24
26
1 2 3 4 5 6 7 8 9 10 11 12 13 14

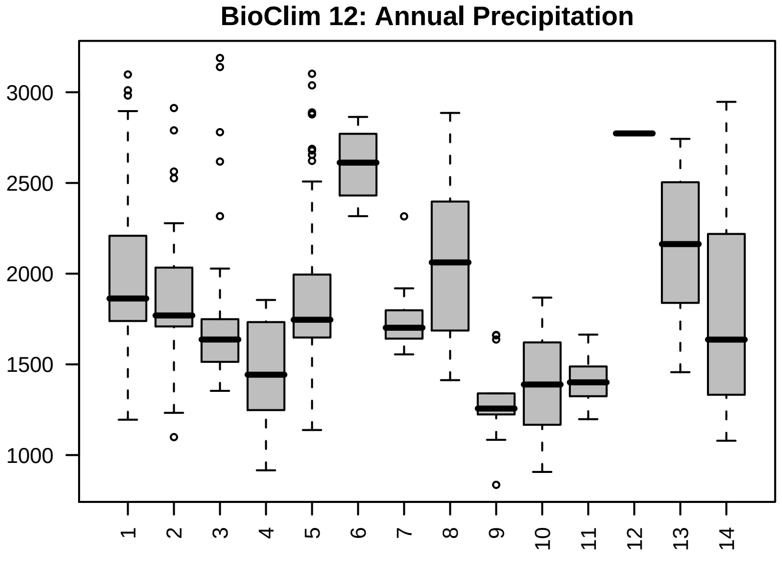
BioClim 12: Annual Precipitation
1000
1500
2000
2500
3000
1 2 3 4 5 6 7 8 9 10 11 12 13 14
