# Supplementary material for: Little ecological divergence associated with speciation in two African rain forest tree genera
Source: BMC Evol Biol. 2011 Oct 11;11:296. doi: 10.1186/1471-2148-11-296 (PMC3203876; doi:10.1186/1471-2148-11-296)
Supplement: Additional file 3 — Variation of bioclim variables BC13-18 for Isolona. Indicates the variation of bioclim variables BC7 to 12 for all sampled species in Isolona. West/Central African species: 1: Isolona congolana; 2: I. hexaloba; 3: I. pleurocarpa; 4: I. zenkeri; 5: I. campanulata; 6: I. cooperi; 7: I. dewevrei; 8: I. thonneri; 9: I. cauliflora. East African species: 10: I. heinsenii; 11: I. linearis. Malagasy species: 12: I. capuroni; 13: I. ghesquierei; 14: I. perrierii. [file 1471-2148-11-296-S3.PDF]

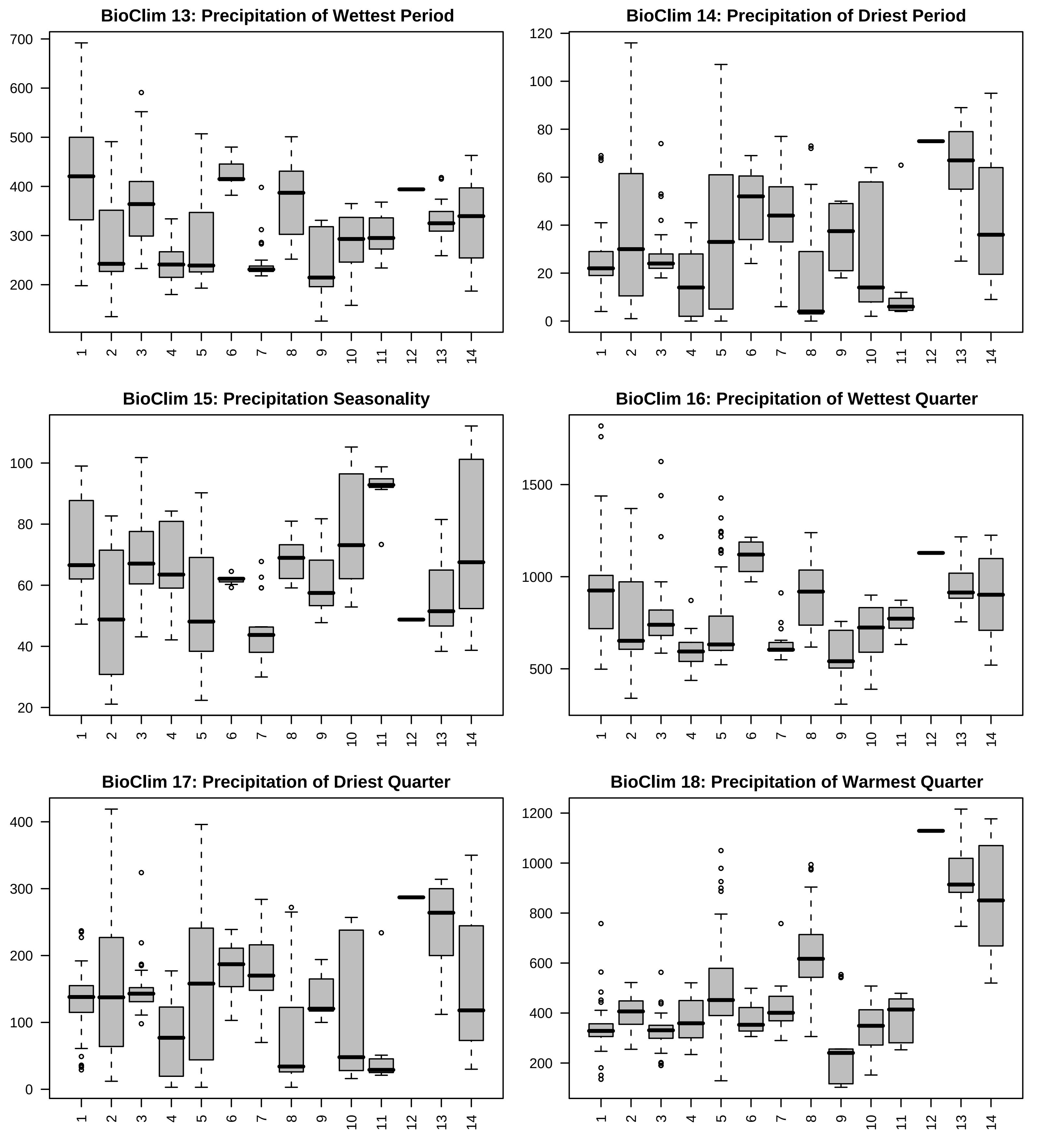
BioClim 13: Precipitation of Wettest Period
700
600
500
400
300
200
1 2 3 4 5 6 7 8 9 10 11 12 13 14
BioClim 14: Precipitation of Driest Period
120
100
80
60
40
20
0
1 2 3 4 5 6 7 8 9 10 11 12 13 14
BioClim 15: Precipitation Seasonality
100
80
60
40
20
1 2 3 4 5 6 7 8 9 10 11 12 13 14
BioClim 16: Precipitation of Wettest Quarter
1500
1000
500
1 2 3 4 5 6 7 8 9 10 11 12 13 14
BioClim 17: Precipitation of Driest Quarter
400
300
200
100
0
1 2 3 4 5 6 7 8 9 10 11 12 13 14
BioClim 18: Precipitation of Warmest Quarter
1200
1000
800
600
400
200
1 2 3 4 5 6 7 8 9 10 11 12 13 14
