# Supplementary figures and images for: Little ecological divergence associated with speciation in two African rain forest tree genera
Source: BMC Evol Biol. 2011 Oct 11;11:296. doi: 10.1186/1471-2148-11-296 (PMC3203876; doi:10.1186/1471-2148-11-296)

BioClim 19: Precipitation of Coldest Quarter

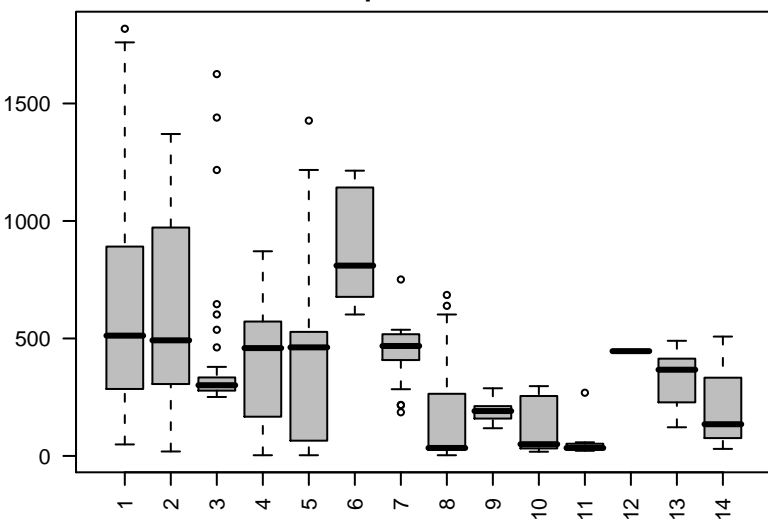

Supplement: Additional file 4 — Variation of bioclim variable BC19 for Isolona. Indicates the variation of bioclim variable BC19 for all sampled species in Isolona. West/Central African species: 1: Isolona congolana; 2: I. hexaloba; 3: I. pleurocarpa; 4: I. zenkeri; 5: I. campanulata; 6: I. cooperi; 7: I. dewevrei; 8: I. thonneri; 9: I. cauliflora. East African species: 10: I. heinsenii; 11: I. linearis. Malagasy species: 12: I. capuroni; 13: I. ghesquierei; 14: I. perrierii. [file 1471-2148-11-296-S4.PDF]

BioClim 19: Precipitation of Coldest Quarter

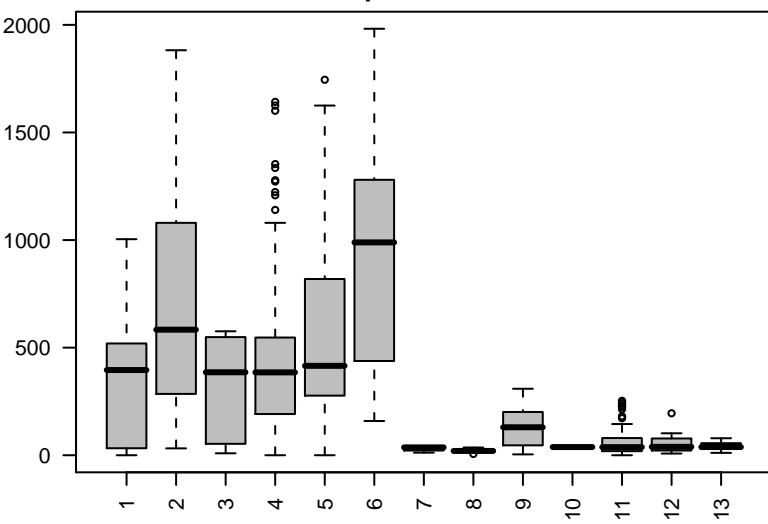

Supplement: Additional file 8 — Variation of bioclim variable BC19 for Monodora. Indicates the variation of bioclim variable BC19 for all sampled species in Monodora. West/Central African species 1: Monodora angolensis 2: M. crispata, 3: M. laurentii, 4: M. myristica, 5: M. tenuifolia, 6: M. undulata. East African species: 7: M. carolinae, 8: M. globiflora, 9: M. grandidieri, 10: M. hastipetala, 11: M. junodii, 12: M. minor, 13: M. stenopetala. [file 1471-2148-11-296-S8.PDF]

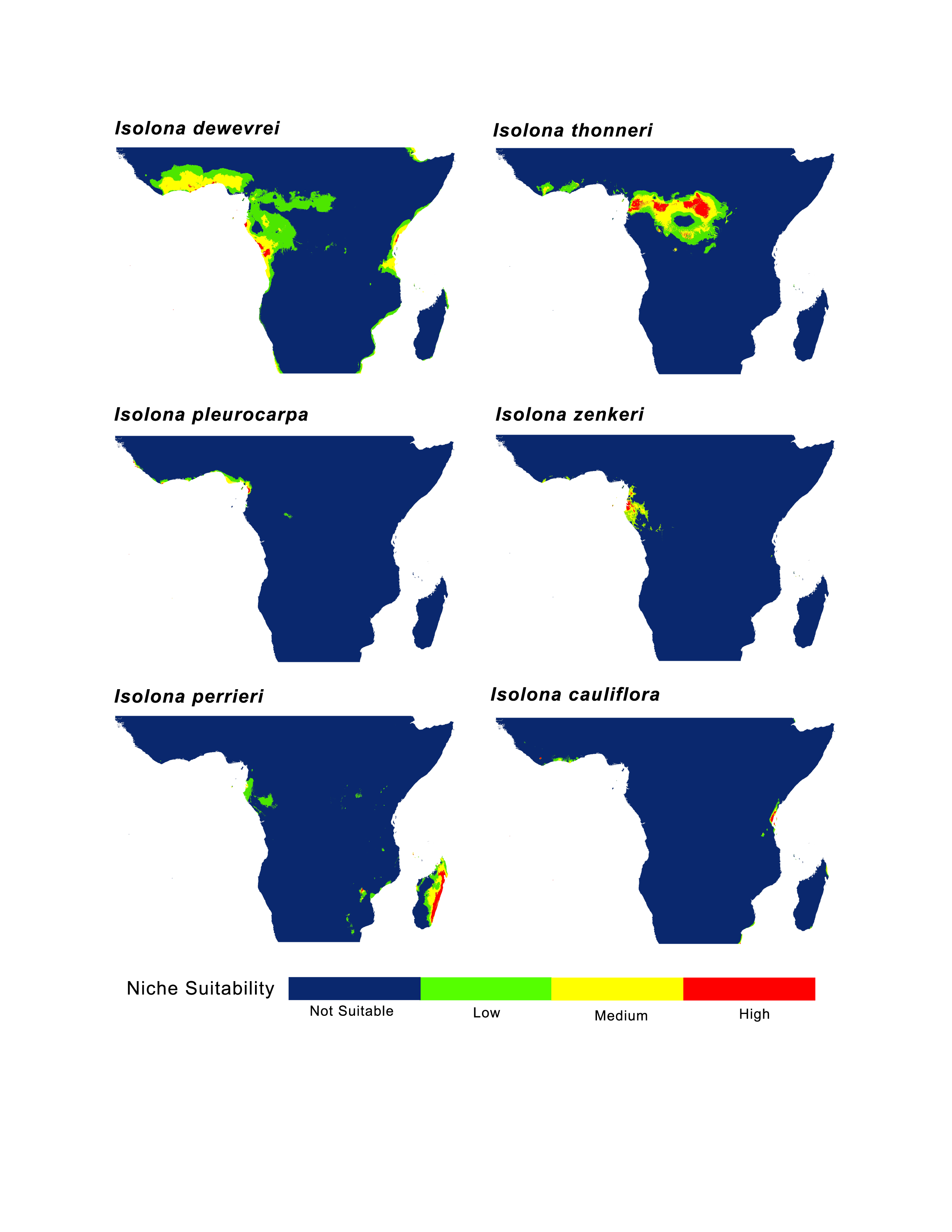

Supplement: Additional file 10 — Potential distribution of Isolona species. Shows the rest of the models generated for Isolona species. [file 1471-2148-11-296-S10.TIFF]

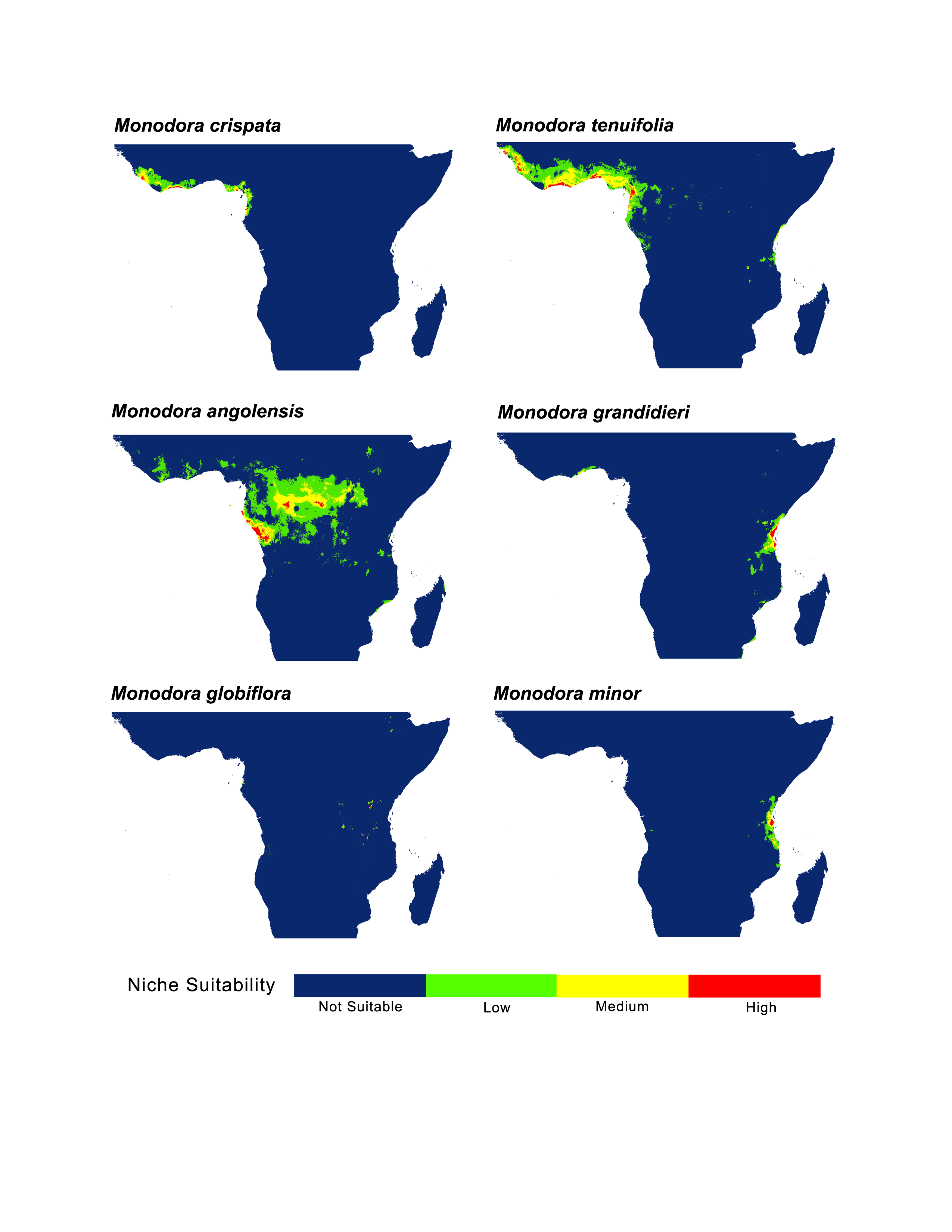

Supplement: Additional file 11 — Potential distribution of Monodora species. Shows the rest of the models generated for Monodora species. [file 1471-2148-11-296-S11.TIFF]

*Isolona ghesquierei*

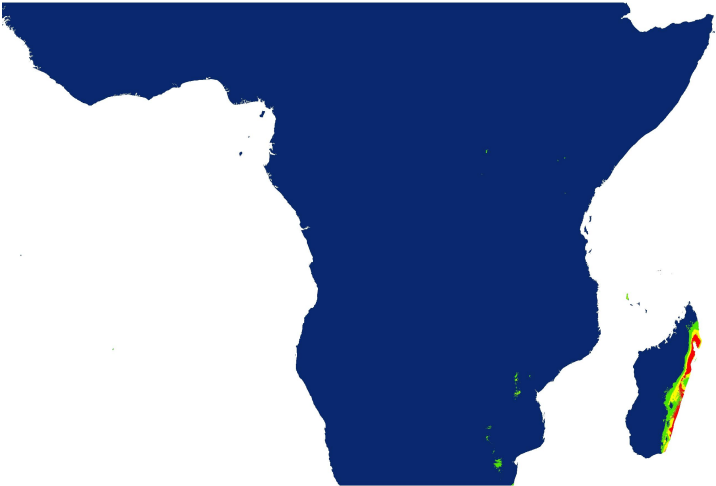

*Monodora laurentii*

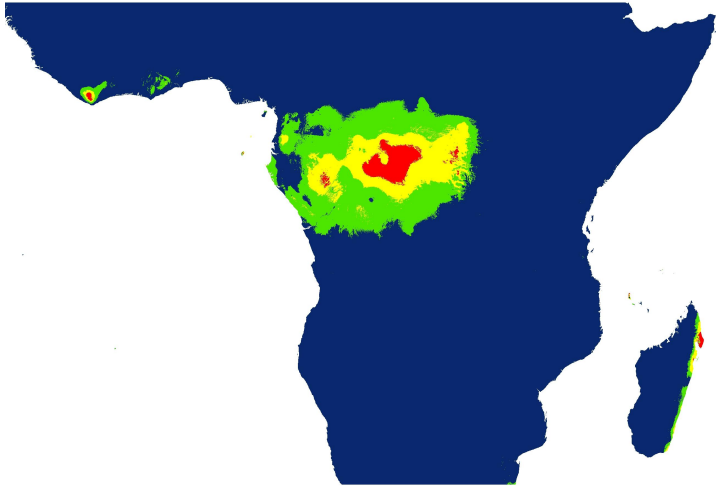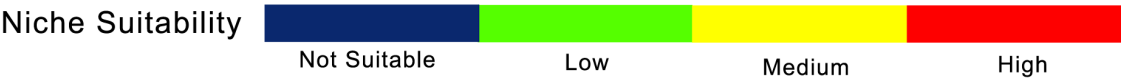

Supplement: Additional file 13 — Distribution of species in Isolona. Shows the geographical location of all data points for each species used in this study. [file 1471-2148-11-296-S13.PDF]

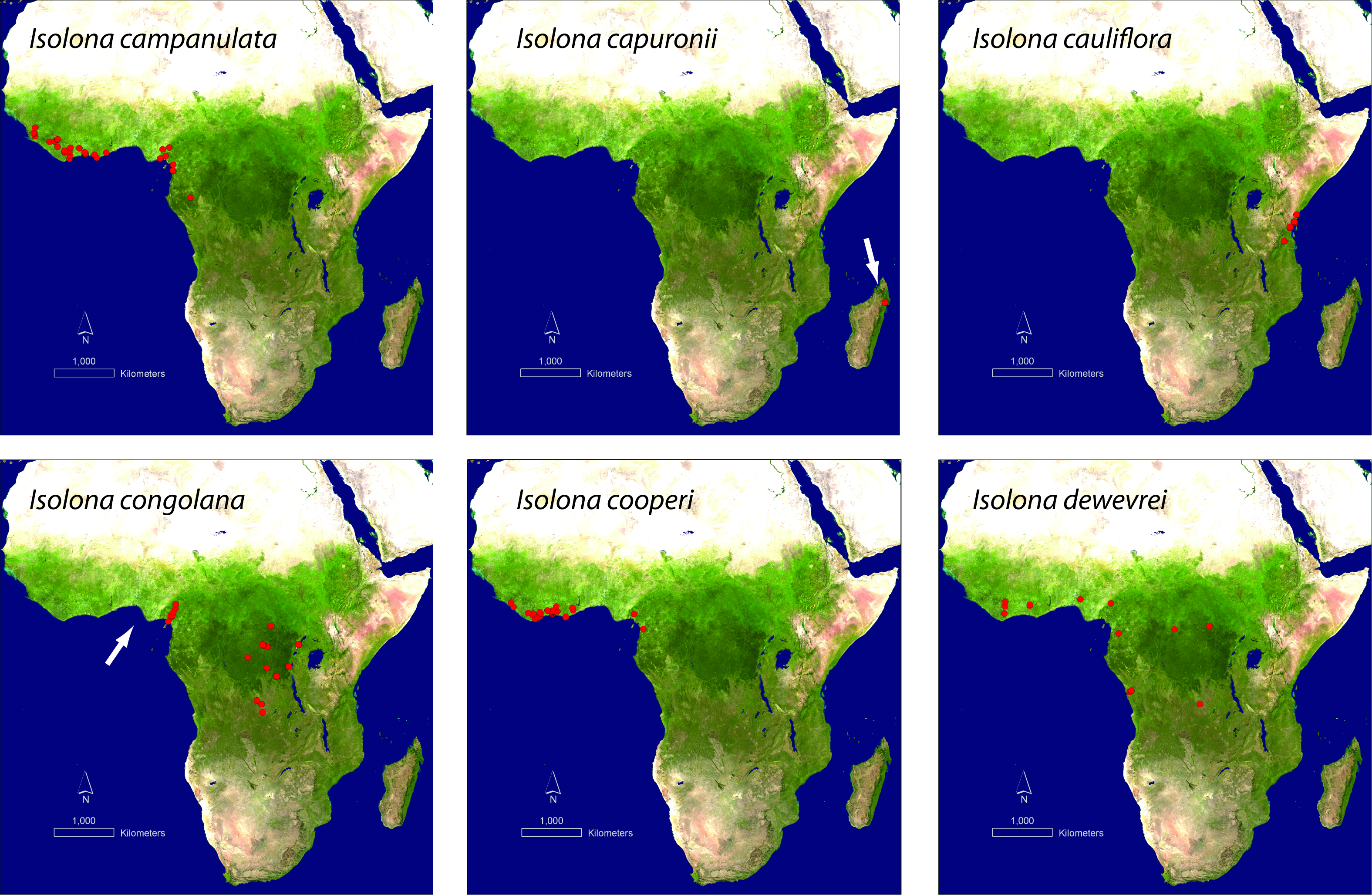

Supplement: Additional file 14 — Distribution of species in Isolona. Shows the geographical location of all data points for each species used in this study. [file 1471-2148-11-296-S14.TIFF]

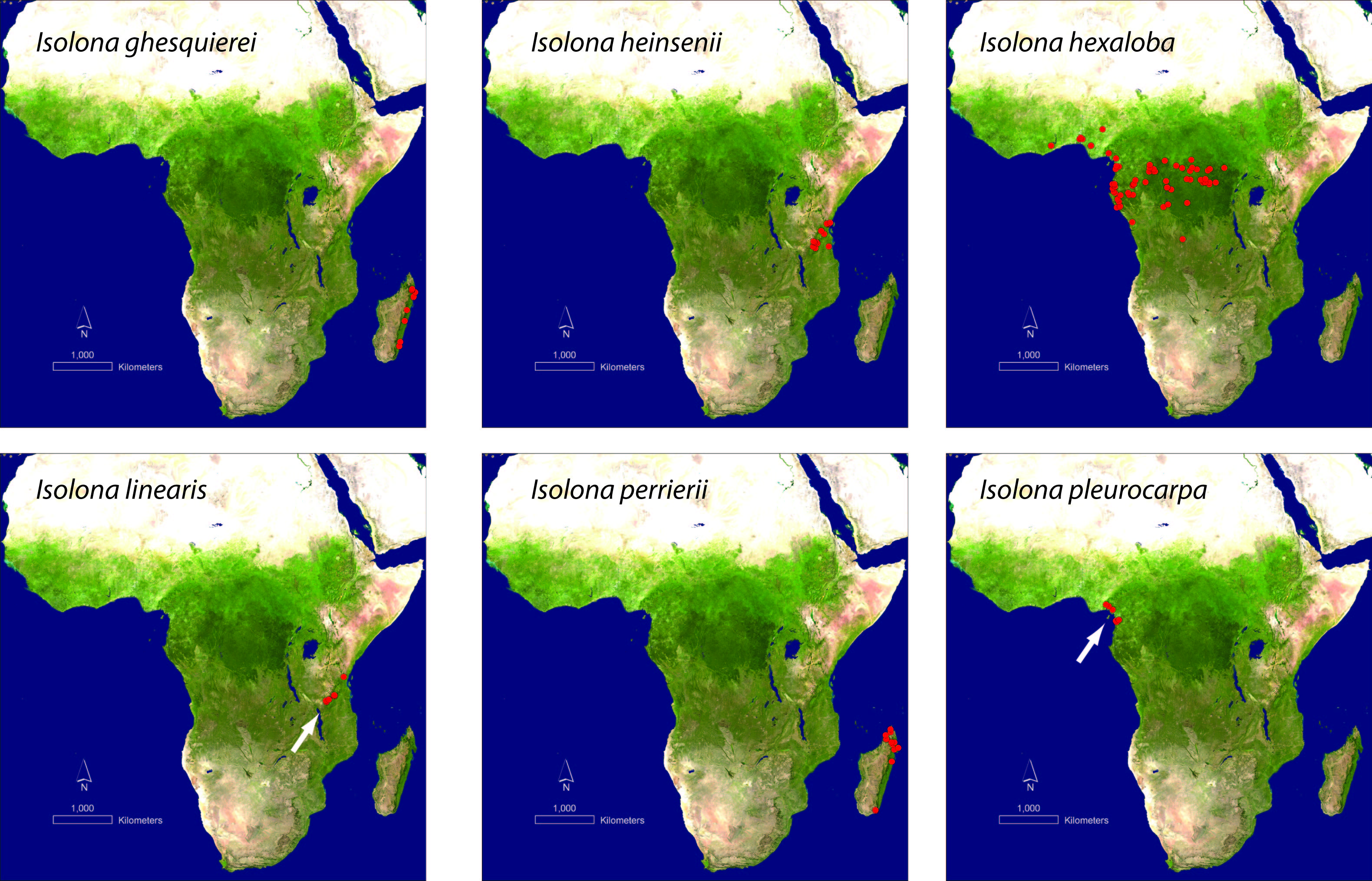

Supplement: Additional file 15 — Distribution of species in Isolona (continue from sup file 1) and Monodora. Shows the geographical location of all data points for each species used in this study. [file 1471-2148-11-296-S15.JPEG]

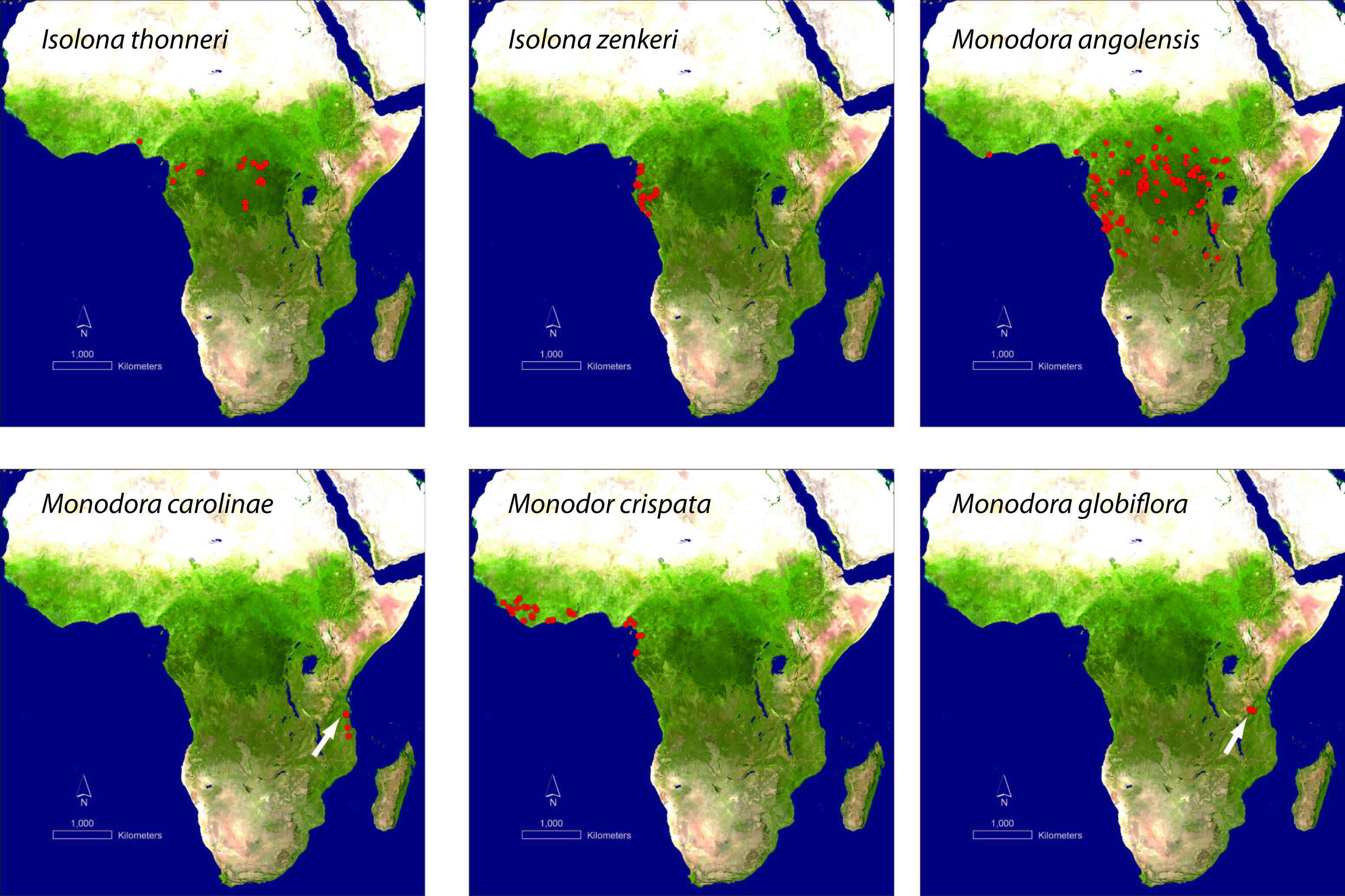

Supplement: Additional file 16 — Distribution of species in Monodora (continue from sup file 2). Shows the geographical location of all data points for each species used in this study. [file 1471-2148-11-296-S16.JPEG]

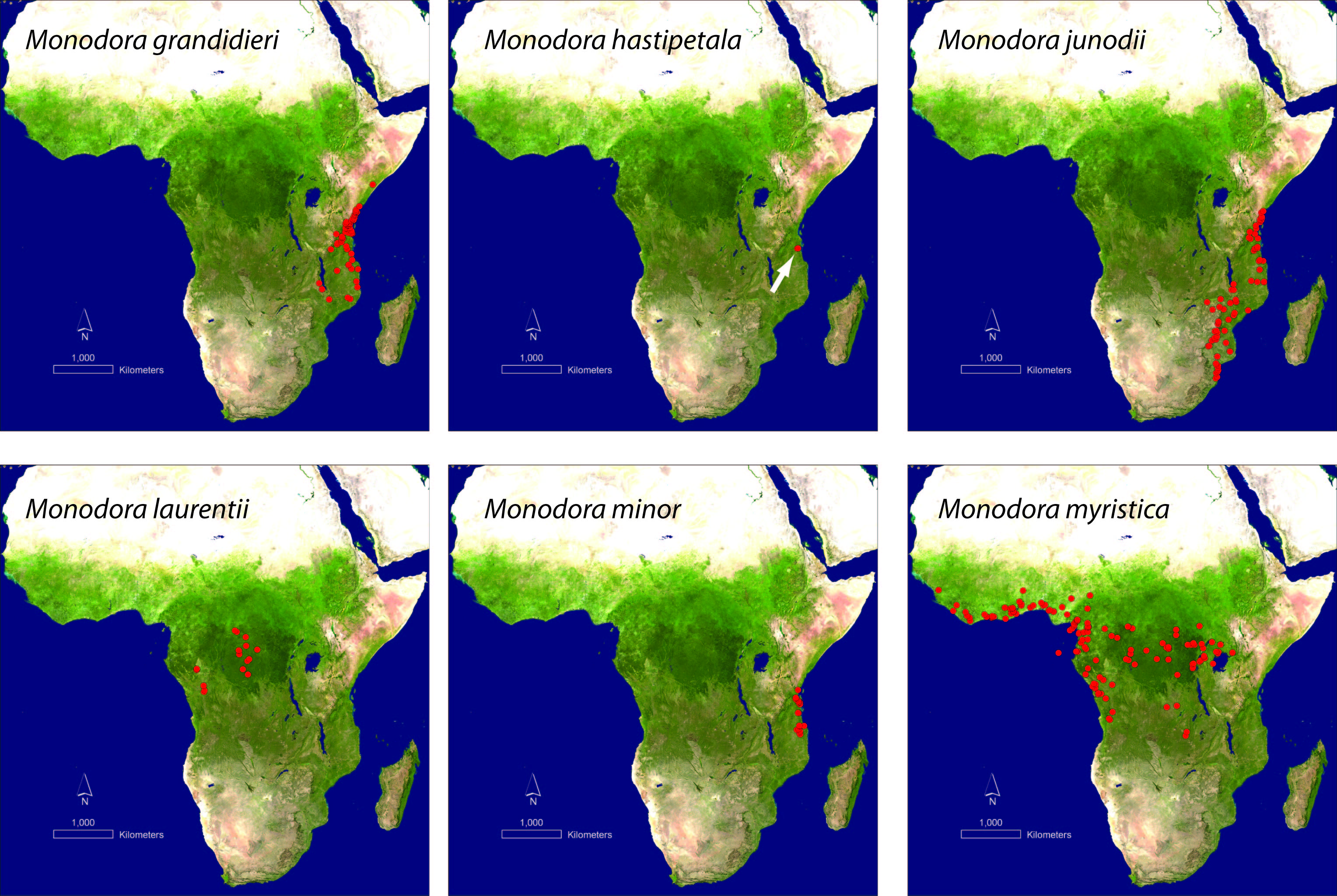

Supplement: Additional file 17 — Distribution of species in Monodora (continue from sup file 3). Shows the geographical location of all data points for each species used in this study. [file 1471-2148-11-296-S17.JPEG]

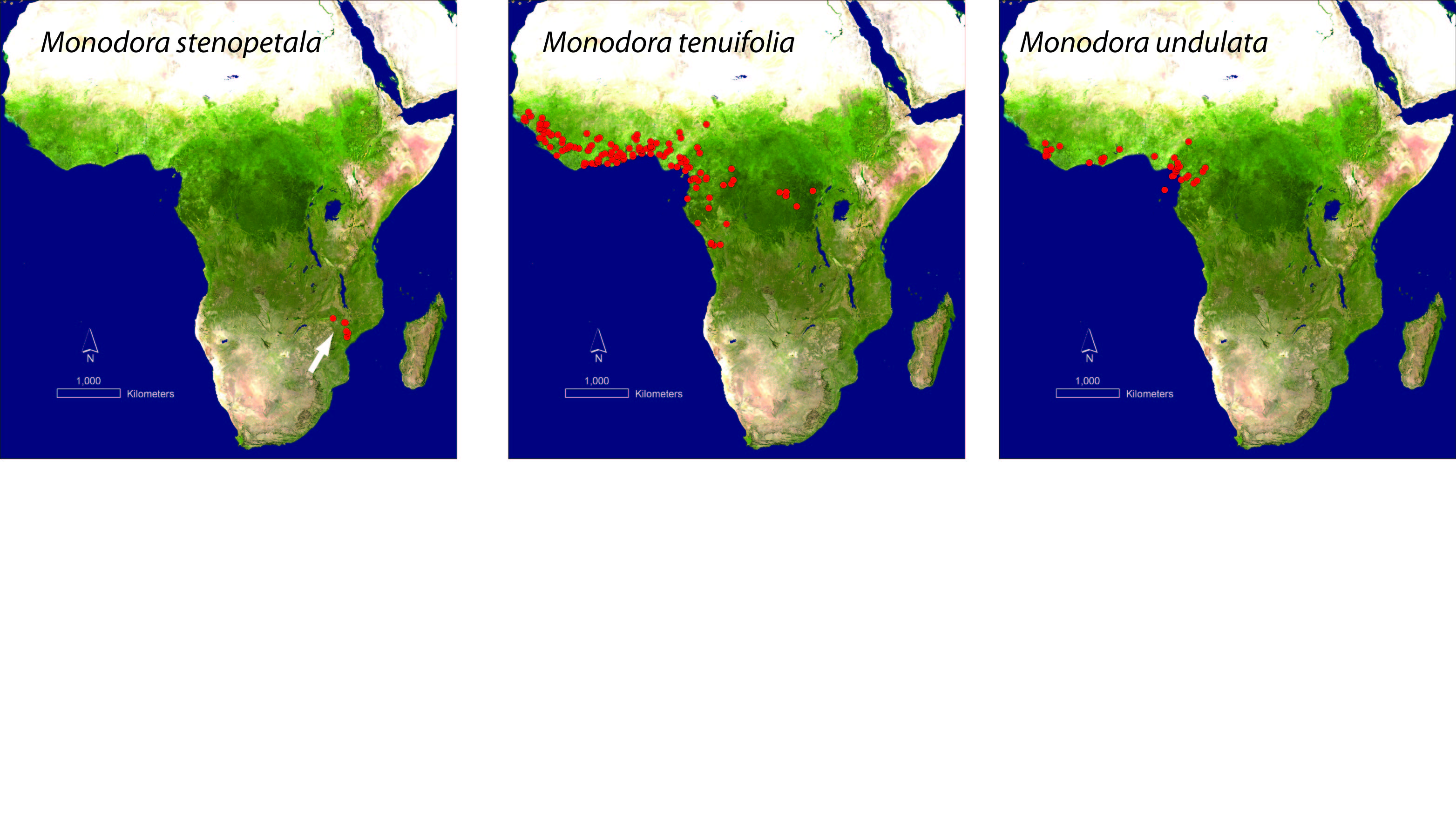

Supplement: Additional file 18 — Distribution of species in Monodora (continue from sup file 4). Shows the geographical location of all data points for each species used in this study. [file 1471-2148-11-296-S18.JPEG]
