# Supplementary material for: Little ecological divergence associated with speciation in two African rain forest tree genera
Source: BMC Evol Biol. 2011 Oct 11;11:296. doi: 10.1186/1471-2148-11-296 (PMC3203876; doi:10.1186/1471-2148-11-296)
Supplement: Additional file 5 — Variation of bioclim variables BC1-6 for Monodora. Indicates the variation of bioclim variables BC1 to 6 for all sampled species in Monodora. West/Central African species 1: Monodora angolensis 2: M. crispata, 3: M. laurentii, 4: M. myristica, 5: M. tenuifolia, 6: M. undulata. East African species: 7: M. carolinae, 8: M. globiflora, 9: M. grandidieri, 10: M. hastipetala, 11: M. junodii, 12: M. minor, 13: M. stenopetala. [file 1471-2148-11-296-S5.PDF]

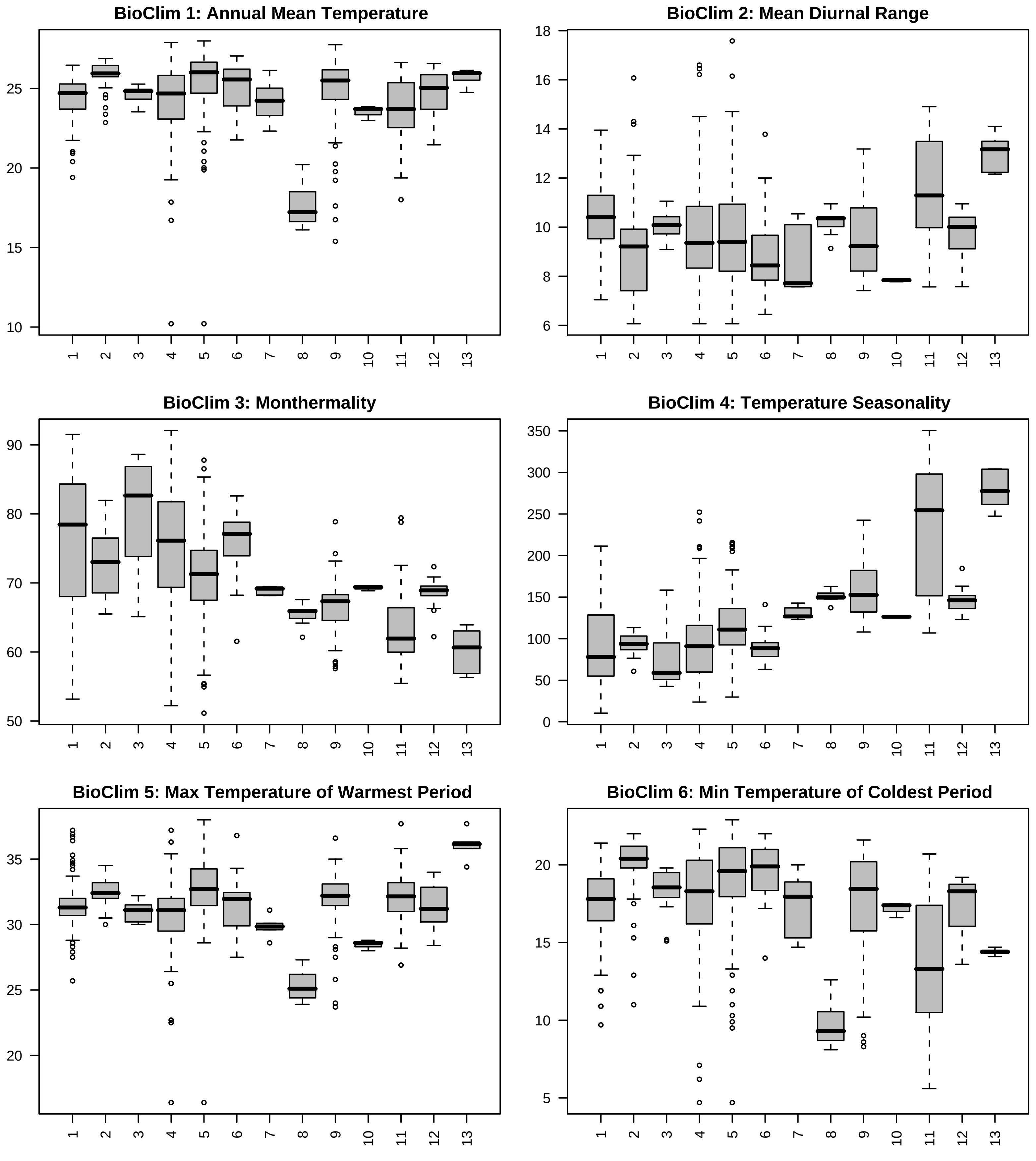
BioClim 1: Annual Mean Temperature
10
15
20
25
1 2 3 4 5 6 7 8 9 10 11 12 13
BioClim 2: Mean Diurnal Range
6
8
10
12
14
16
18
1 2 3 4 5 6 7 8 9 10 11 12 13
BioClim 3: Monthermality
50
60
70
80
90
1 2 3 4 5 6 7 8 9 10 11 12 13
BioClim 4: Temperature Seasonality
0
50
100
150
200
250
300
350
1 2 3 4 5 6 7 8 9 10 11 12 13
BioClim 5: Max Temperature of Warmest Period
20
25
30
35
1 2 3 4 5 6 7 8 9 10 11 12 13
BioClim 6: Min Temperature of Coldest Period
5
10
15
20
1 2 3 4 5 6 7 8 9 10 11 12 13
