# Supplementary material for: Little ecological divergence associated with speciation in two African rain forest tree genera
Source: BMC Evol Biol. 2011 Oct 11;11:296. doi: 10.1186/1471-2148-11-296 (PMC3203876; doi:10.1186/1471-2148-11-296)
Supplement: Additional file 6 — Variation of bioclim variables BC7-12 for Monodora. Indicates the variation of bioclim variables BC7 to 12 for all sampled species in Monodora. West/Central African species 1: Monodora angolensis 2: M. crispata, 3: M. laurentii, 4: M. myristica, 5: M. tenuifolia, 6: M. undulata. East African species: 7: M. carolinae, 8: M. globiflora, 9: M. grandidieri, 10: M. hastipetala, 11: M. junodii, 12: M. minor, 13: M. stenopetala. [file 1471-2148-11-296-S6.PDF]

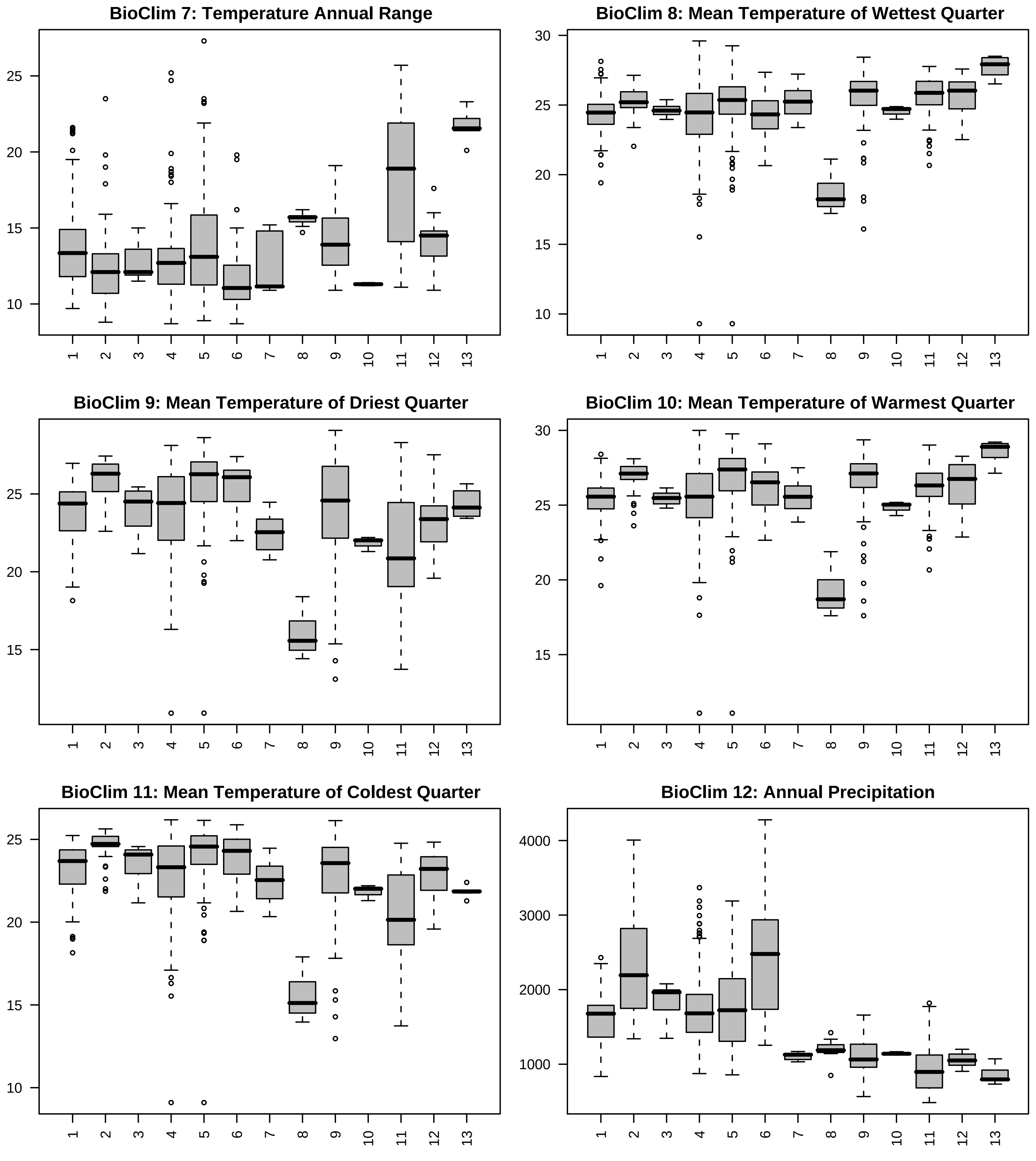
BioClim 7: Temperature Annual Range
10
15
20
25
1 2 3 4 5 6 7 8 9 10 11 12 13
BioClim 8: Mean Temperature of Wettest Quarter
10
15
20
25
30
1 2 3 4 5 6 7 8 9 10 11 12 13
BioClim 9: Mean Temperature of Driest Quarter
15
20
25
1 2 3 4 5 6 7 8 9 10 11 12 13
BioClim 10: Mean Temperature of Warmest Quarter
15
20
25
30
1 2 3 4 5 6 7 8 9 10 11 12 13
BioClim 11: Mean Temperature of Coldest Quarter
10
15
20
25
1 2 3 4 5 6 7 8 9 10 11 12 13
BioClim 12: Annual Precipitation
1000
2000
3000
4000
1 2 3 4 5 6 7 8 9 10 11 12 13
