# Supplementary material for: Little ecological divergence associated with speciation in two African rain forest tree genera
Source: BMC Evol Biol. 2011 Oct 11;11:296. doi: 10.1186/1471-2148-11-296 (PMC3203876; doi:10.1186/1471-2148-11-296)
Supplement: Additional file 7 — Variation of bioclim variables BC13-18 for Monodora. Indicates the variation of bioclim variables BC7 to 12 for all sampled species in Monodora. West/Central African species 1: Monodora angolensis 2: M. crispata, 3: M. laurentii, 4: M. myristica, 5: M. tenuifolia, 6: M. undulata. East African species: 7: M. carolinae, 8: M. globiflora, 9: M. grandidieri, 10: M. hastipetala, 11: M. junodii, 12: M. minor, 13: M. stenopetala. [file 1471-2148-11-296-S7.PDF]

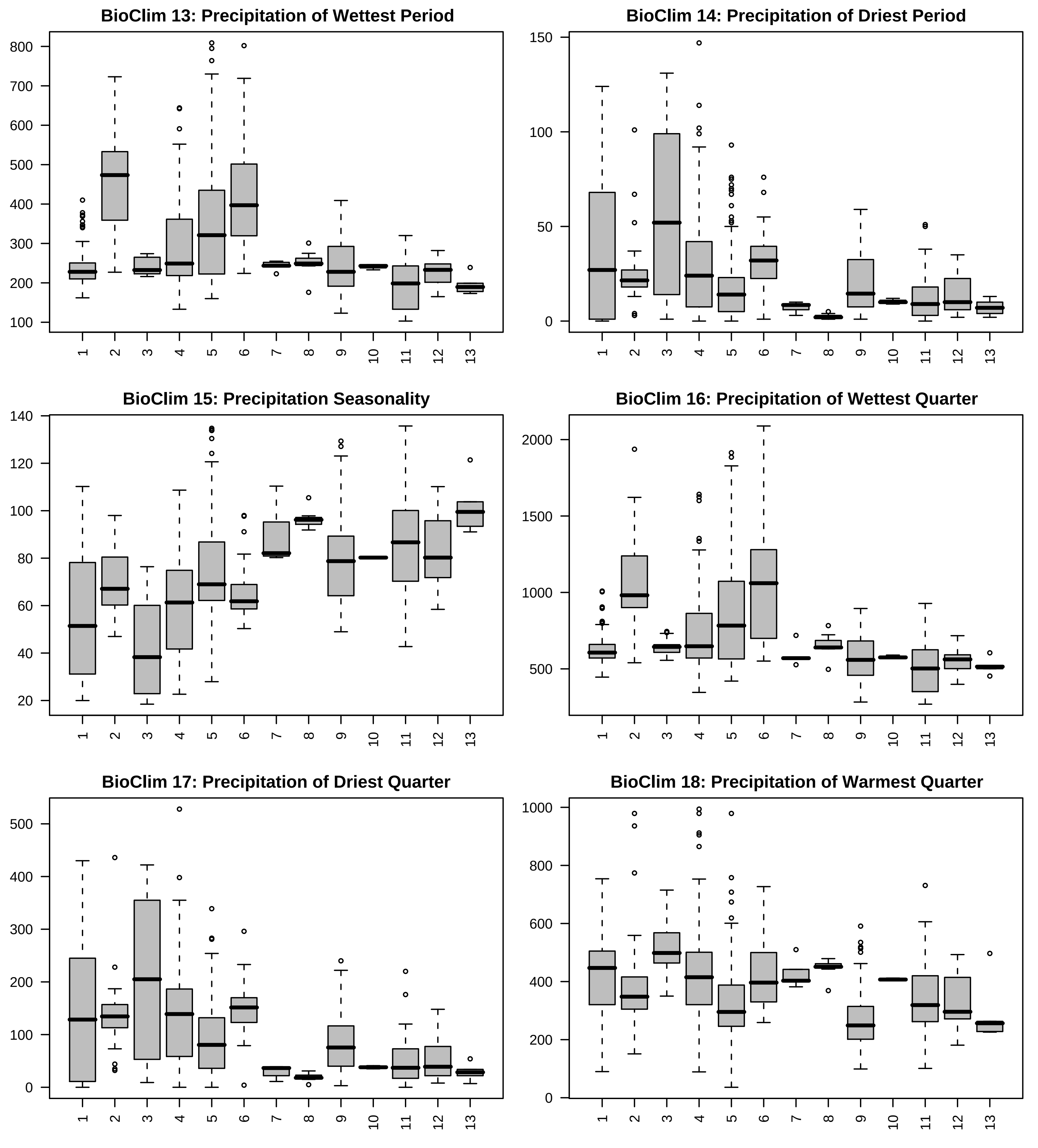
BioClim 13: Precipitation of Wettest Period
100
200
300
400
500
600
700
800
BioClim 14: Precipitation of Driest Period
0
50
100
150
BioClim 15: Precipitation Seasonality
20
40
60
80
100
120
140
BioClim 16: Precipitation of Wettest Quarter
500
1000
1500
2000
BioClim 17: Precipitation of Driest Quarter
0
100
200
300
400
500
BioClim 18: Precipitation of Warmest Quarter
0
200
400
600
800
1000
1 2 3 4 5 6 7 8 9 10 11 12 13
